# Retroelement co-option disrupts the cancer transcriptional programme

**DOI:** 10.1186/s13073-025-01479-9

**Published:** 2025-05-07

**Authors:** Jane Loong, Rachael Thompson, Callum Hall, Laura Doglio, Judith Pape, Tobias Plowman, George Kassiotis

**Affiliations:** 1 Retroviral Immunology Laboratory, The Francis Crick Institute, 1 Midland Road, London, NW1 1 AT UK; 2 Department of Infectious Disease, Faculty of Medicine, Imperial College London, London, UK

**Keywords:** Cancer, Retrotransposable elements, Isoform switching, Transcriptional disruption

## Abstract

**Background:**

Transcriptional activation of otherwise repressed retrotransposable elements (RTEs) is a hallmark of cancer, shaping tumour progression and immunogenicity by multifaceted, yet incompletely understood, mechanisms.

**Methods:**

We used an extended pan-cancer transcriptome assembly to identify potential effects of RTEs on the genes within which they have integrated or those in proximity. These were subsequently verified in test cases by further analysis of transcriptional profiles in cancer patient data, and by in vitro studies involving restoration of gene activity, and proliferation and migration assays in cancer cell lines.

**Results:**

We report that cancer-specific transcriptional activation of RTEs causes frequent reduction or loss of gene function. Exonisation and alternative splicing of RTEs creates non-functional RNA and protein isoforms and derepressed RTE promoter activity initiates antisense transcription, both at the expense of the canonical isoforms. Contrary to theoretical expectation, transcriptionally activated RTEs affect genes with established tumour-promoting functions, including the common essential *RNGTT* and the lung cancer-promoting *CHRNA5* genes. Furthermore, the disruptive effect of RTE activation on adjacent tumour-promoting genes is associated with slower disease progression in clinical data, whereas experimental restoration of gene activity enhances tumour cell growth and invasiveness in vitro.

**Conclusions:**

These findings underscore the gene-disruptive potential of seemingly innocuous germline RTE integrations, unleashed only by their transcriptional utilisation in cancer. They further suggest that such metastable RTE integrations are co-opted as sensors of the epigenetic and transcriptional changes occurring during cellular transformation and as executors that disrupt the function of tumour-promoting genes.

**Supplementary Information:**

The online version contains supplementary material available at 10.1186/s13073-025-01479-9.

## Background

Similar to all mammalian genomes, the human genome has amassed over 4 million recognisable integrations of retrotransposable elements (RTEs) of diverse subfamilies, some of which are primate-specific, such as the human endogenous retrovirus (HERV) H subfamily of long terminal repeat (LTR) RTEs and the *Alu* subfamily of non-LTR RTEs [1]. While the vast majority of human RTEs have lost the ability to retrotranspose, new germline integrations of active RTEs are acquired slowly but continually [2].

RTE integration poses a significant risk of insertional mutagenesis, which is higher for the recently acquired or active subfamilies. Highly deleterious mutations may be quickly counterselected, whereas less damaging integrations are retained for longer evolutionary times. The genetic diversity generated by RTE integration, as well as the regulatory sequences they carry, can be co-opted in the evolution of new physiological host functions and transcriptional networks [3, 4]. Indeed, RTE enhancer and promoter activities are co-opted in the regulatory networks of placentation or the interferon (IFN) response genes [5], and alternative RTE exons in the diversification of protein isoforms and function [4]. The repetitive nature of RTEs provides templates for non-allelic homologous recombination, leading to genomic rearrangements and gene fusions, which in turn may create alternative or novel protein isoforms with selectable function in physiology or in cancer [6, 7].

In addition to evolutionary selection, epigenetic control of RTEs further mitigates the potentially damaging effect of RTE integrations on host gene function by preventing their independent transcription or inclusion in host gene transcripts [8]. Epigenetic repression acts faster than evolutionary processes and allows for the latter to determine the ultimate fate of a given RTE integration. However, epigenetic repression is reversible and can be lost, particularly following the major epigenetic changes seen in cancer [8]. In turn, RTE release from epigenetic control may allow for previously hidden effects on the gene near or within which they have integrated to manifest.

Altered transcriptional activity of RTEs has been consistently observed in most cancer types, associated with substantial effects on the cancer transcriptome [8, 9]. RTE transcriptional inclusion in cancer results in aberrant transcription and splicing patterns, often in a cancer type-specific fashion [10]. However, given its high degree of complexity, the functional consequences of the aberrant cancer transcriptome created by RTE derepression are not yet fully understood. RTEs can be co-opted in driving elevated or ectopic expression of genes with tumour-promoting, or in creating tumorigenic protein isoforms. Instances of such onco-exaptation events include the elevated expression of *CSF1R* and *IRF5* in Hodgkin’s lymphoma [11, 12], the ectopic expression of *CALB1* (encoding calbindin) in squamous lung cancer [13], and the creation of an oncogenic form of ALK (anaplastic lymphoma kinase) in melanoma [14]. Moreover, comprehensive analysis of epigenetically reactivated RTE promoter activity has identified > 100 potential onco-exaptation events involving oncogenes in diverse cancer types [15].

Owing to the increase in tumour cell fitness they confer, RTE-mediated activation of tumour-promoting genes would be increasingly enriched over the course of tumour progression and molecular evolution, making such onco-exaptation events easier to identify in cancer transcriptomes. In contrast, effects of epigenetically reactivated RTEs that compromise the function of an essential gene would also compromise tumour cell fitness and would, therefore, be counterselected during tumour evolution, giving the appearance of rarer occurrence. However, the potential of transcriptionally reactivated RTEs to disrupt the function of tumour-promoting genes may be a selectable trait in host species evolution.

Here, we investigated the degree to which gene function may be compromised by transcriptionally reactivated RTEs in cancer. We made use of our increasing understanding of the complexity of cancer transcriptomes and identified cancer-specific transcript isoforms, created by transcriptional inclusion of RTEs at the expense of the protein-coding canonical isoform of adjacent genes. Counterintuitively, many of the affected genes have an established tumour-promoting role, and, in test cases, restoration of their expression accelerates tumour cell-intrinsic growth and invasiveness.

## Methods

### Publicly available RNA-sequencing (RNA-seq) samples used in this study

RNA-seq samples (poly(A) selected RNA), representing 31 primary and one metastatic indications (*n* = 24 per indication), were obtained from the Cancer Genome Atlas Program (TCGA) [16], as previously described [10]. For further validation, additional samples were downloaded for specific cancer indications and matching normal tissues (COAD, colon adenocarcinoma *n* = 239; EAC, oesophageal adenocarcinoma *n* = 78; KIRC, kidney renal clear cell carcinoma *n* = 538; LUAD, lung adenocarcinoma *n* = 419; LUSC, lung squamous cell carcinoma *n* = 362; MESO, mesothelioma *n* = 24; OV, ovarian serous cystadenocarcinoma *n* = 419; primary SKCM, skin cutaneous melanoma *n* = 101; metastatic SKCM *n* = 224; normal colon *n* = 39; normal oesophagus *n* = 9; normal kidney *n* = 71; normal lung *n* = 24; normal ovary *n* = 12; normal skin *n* = 36). Additional RNA-seq samples from normal tissues were obtained from the Genotype-Tissue Expression (GTEx) Project [17] (*n* = 2–156 per tissue type), as previously described [10]. The data files were downloaded from the database of Genotypes and Phenotypes (dbGaP) [18] accession numbers phs000178.v10.p8.c1 and phs000424.v7.p2.c1. RNA-seq samples from cancer cell lines (*n* = 933) were downloaded from the Cancer Cell Line Encyclopedia (CCLE) [19]. Other publicly available dataset supporting the findings of this study included the following: RNA-seq data from a renal cell carcinoma cell line RCC4 with restored expression of the von Hippel-Lindau (VHL) tumour suppressor protein (GSE120887) [20]; ISO-seq data from ESCC cell line TE5 and normal immortalised oesophageal squamous epithelial cell line SHEE (PRJNA515570) [21]; long-read RNA-seq data from HEK293 T and A549 cells [22]. Details of sample collection, ethics approvals and metadata can be found each respective study.

### RNA preparation and sequencing

For bulk RNA-seq of A498 cells with deletion in *HERVE 6q15*, RNA was extracted from cell lines using RNeasy kit (Qiagen, Cat #74,104) and library prep was performed with NEBNext Ultra II Directional PolyA mRNA kit (NEB, Cat #E7760). Samples were then sequenced on a NovaSeq (Illumina). Raw data were deposited at the EMBL-EBI repository (E-MTAB-14514) [23].

### Transcript identification, read mapping and quantitation

Samples from TCGA were downloaded through the *gdc-client* application as.bam files, which were subsequently parsed with a custom Bash pipeline using GNU parallel [24], and converted to.fasta files using SAMtools v1.8 [25]. RNA-seq data from TCGA, GTEx, CCLE and listed previous studies were mapped to our de novo cancer transcriptome assembly and counted as previously described [10]. Briefly, transcripts per million (TPM) values were calculated for all transcripts in the transcript assembly [10] with a custom Bash pipeline and Salmon v0.8.2 [26], which uses a probabilistic model for assigning reads aligning to multiple transcript isoforms, based on the abundance of reads unique to each isoform [26]. Read count tables were additionally imported into Qlucore Omics Explorer v3.9 (Qlucore, Lund, Sweden) for further downstream expression analyses and visualisation. Splice junctions were visualised using the Integrative Genome Viewer (IGV) v2.4.19 [27]. Long-read RNA-seq samples were aligned to the GRCh38/hg38 human genome using minimap2 v2.17 [28]. For assemblies of long-read RNA-seq reads, the obtained.bam files were first converted to bed12 using bam2bed12.py script from FLAIR suit [29]. High-confidence isoforms were selected using “collapse” function from flair.py script [29].

### Hypoxia scores

The hypoxia scores [30] for all TCGA samples were kindly provided by Prof. David Mole (Nuffield Department of Medicine, University of Oxford). The hypoxia scores for KIRC samples were filtered; however, mismatches in sample names due to updates by TCGA to the naming system of files meant the mean hypoxia score per patient was used here. Of the 479 KIRC patients with hypoxia scores calculated for tumour samples, 406 had hypoxia scores for one sample, 70 for two samples, and three patients had the mean hypoxia score calculated from three samples.

### Repeat annotation and enrichment analysis

Repeat regions were annotated as previously described [31]. Briefly, hidden Markov models (HMMs) representing known human repeat families (Dfam 2.0 library v150923) were used to annotate GRCh38 using RepeatMasker, configured with nhmmer. RepeatMasker annotates LTR and internal regions separately; thus, tabular outputs were parsed to merge adjacent annotations for the same element. Dfam 2.0 differs from later releases by 81 out of 1407 repeat families, with 97 families added and 16 families removed in Dfam 3.8. These differences are partly due to reclassification of existing families and to inclusion of certain low-copy composite repeats. Enrichment analysis of repeat types was performed using Fisher’s exact test in MATLAB (version R2022b, TheMathWorks), followed by the Bonferroni-Holm method to correct for multiple testing. The RTE content, as well as RTE exonisation in specific loci of interest, was further manually inspected and validated on IGV using the Telomere-to-Telomere (T2 T)-CHM13 genome sequence [32] and the Dfam 3.6 release.

### Functional gene annotation by gene ontology

Pathway analyses were performed using g:Profiler [33] with genes ordered by the degree of differential expression. *p* values were estimated by hypergeometric distribution tests and adjusted by multiple testing correction using the g:SCS (set counts and sizes) algorithm, integral to the g:Profiler server [33].

### Survival analysis and hazard ratio calculations

Overall survival time was downloaded for TCGA data alongside all other metadata. Survival analysis was run using R and RStudio (version 2023.12.0 Build 369). Univariate and multivariate analyses, hazard ratio calculations and survival curve plotting were performed using GraphPad Prism (version 10.3).

### Comparative genomics and sequence alignments

Multiple genomic sequence alignments were carried out using the comparative genomic tool from Ensembl [34]. For sequence divergence of the *HERVE 6q15* and *HERVH Xp22.2* proviruses, the following primate species were compared: *Homo sapiens* (human), *Pan troglodytes* (chimp), *Pan paniscus* (bonobo), *Gorilla gorilla* (gorilla), *Pongo abelii* (orangutan) and *Nomascus leucogenys* (gibbon). Absolute complexity scores were calculated using Vector NTI 11.5.

### Cell lines

All cell lines were obtained from the Cell Services facility of The Francis Crick Institute and verified as mycoplasma-free. All human cell lines were further validated by DNA fingerprinting (Table 1).

**Table 1:** Cell lines used in this study

Cell line	RRID	Base media	FBS	L-glutamine	Penicillin	Streptomycin
A498	CVCL_1056	DMEM	10%	–	100 U/mL	0.1 mg/mL
OE19	CVCL_1622	RPMI 1640	10%	2 mM	100 U/mL	0.1 mg/mL
A549	CVCL_0023	DMEM	10%	–	100 U/mL	0.1 mg/mL
HEK293 T	CVCL_0063	IMDM	5%	2 mM	100 U/mL	0.1 mg/mL
HCC4006	CVCL_1269	RPMI 1640	10%	2 mM	100 U/mL	0.1 mg/mL

All base media sourced from Gibco, penicillin–streptomycin solution from Millipore-Sigma (P4333-100ML), L-glutamine from Merck (G7513-100ML), and FBS from Thermo Fisher Scientific

### Cell transfections

HEK293 T and A549 cells were seeded at a density of 200,000 cells/well in 2 mL of culture media 24 h prior transfection in 6-well plates. Cells were then transfected with 5 µg of plasmid each expressing the following sequence (see Tables 2, 3, 4, 5 and 6): CHRNA5 (pcDNA3.1-CHRNA5, Genewiz) or CHRNA5[AluSz] (pcDNA3.1-CHRNA5[AluSz], Genewiz) or CHRNA5-Full intron 5 (pcDNA3.1-CHRNA5-Full intron 5, Genewiz) or CHRNA5-RTE deleted (pcDNA3.1-CHRNA5-RTE deleted, Genewiz) using Lipofectamine 3000 transfection reagent (Thermo Fisher). Cells were then seeded for immunofluorescence staining.

### Polymerase chain reaction (PCR)

PCR was performed using KOD Hot Start Master Mix (Sigma) with the following primers (Table 2):

**Table 2:** PCR primers used in this study

*Gene name*	*Forward*	*Reverse*
HERVE(RNGTT) KO validation	TTCTAAAAGATCATCAGTCAGC	CCACCAAATTACATGCAT
HERVE(RNGTT)−5′ arm specific	TTCTAAAAGATCATCAGTCAGC	GGACTATAACCATTATATGGGA
HERVE(RNGTT)−3′ arm specific	ACTAACAGTACAGATGTACAGATAC	CCACCAAATTACATGCAT
CHRNA5-FL	TGACTATGGTGGAATAAAAG	AAAGCCCAAGAGATCCAA
CHRNA5-SF	TGACTATGGTGGAATAAAAG	GAAGAAGATCTGCATTTGTA

### Reverse transcriptase-based quantitative PCR (RT-qPCR)

RNA was extracted using the RNeasy kit (Qiagen). cDNA was synthesised using the Maxima First Strand cDNA Synthesis Kit (Thermo Fisher), and qPCR performed using Applied Biosystems Fast SYBR Green (Thermo Fisher) using the following primers (Table 3):

**Table 3:** RT-qPCR primers used in this study

*Gene name*	*Forward*	*Reverse*
RNGTT	ACTTGAAGGAAATTTTGCCA	GGCTTCCATTTCAAAATATC
CHRNA5	AGATGGAACCCTGATGACTATGGT	AAACGTCCATCTGCATTATCAAAC
CHRNA5-FL	AAATTCATAGCCCAGGTT	AAAGCCCAAGAGATCCAA
CHRNA5-SF	TCACTCAGAAAGAGGAAACT	GAAGAAGATCTGCATTTGTA
HPRT	TGACACTGGCAAAACAATGCA	GGTCCTTTTCACCAGCAAGCT

Values were normalised to HPRT expression using the ΔC_T_ method

### Cas9-mediated editing

The HERVE provirus in the RNGTT locus was targeted by the following guide RNA (gRNA) sequences (Table 4):

**Table 4:** gRNAs used in this study

*Name*	*Sequence*
RNGTT 5′ arm 1	GAGTATCTGACTGTGACTAA
RNGTT 5′ arm 2	AGACAACTAATATCAAGAGA
RNGTT 3′ arm 1	AGTCAGGTACAAGCCAACAT
RNGTT 3′ arm 2	ACAGATGTACAGATACTTAT

The above gRNA sequences were synthesised by IDT in Alt-R^TM^CRISPR-Cas9 sgRNA form and resuspended in TE buffer. A498 cells were cultured to confluence, passaged and seeded into a 6-well dish at a density of 200,000 cells/well. The next day, all four sgRNAs (300 ng per sgRNA) were resuspended with Lipofectamine Cas9 Plus reagent (12.5 µL) and Alt-R™.p.Cas9-GFP V3 (6250 ng) in OptiMEM (125 µL), mixed with 7.5 µL Lipofectamine CRISPRMAX reagent in 125 µL OptiMEM. Twenty-four hours later, positively transfected cells were single cell sorted based on their green fluorescent protein (GFP) status using a BD FACSAria II (BD Biosciences) onto 96-well plates. After 4–6 weeks, genomic DNA samples were taken from all clones using DNeasy Blood & Tissue Kit (Qiagen). The deleted allele was confirmed with primers—HERVE(RNGTT) KO validation and the wild-type allele was amplified with primers—HERVE(RNGTT)−5′ arm specific and HERVE(RNGTT)−3′ arm specific to confirm homozygous excision of HERVE provirus.

### Amplicon Sanger sequencing

Genomic DNA PCR products from A498 cells were sent to Genewiz for PCR clean-up and Sanger sequencing with the following sequencing primers (Table 5):

**Table 5:** Amplicon Sanger sequencing primers used in this study

*Gene name*	*Forward*	*Reverse*
HERVE(RNGTT) KO validation	TTCTAAAAGATCATCAGTCAGC	CCACCAAATTACATGCAT

### Amplicon next-generation sequencing

Amplicons from HCC4006 cell cDNA specific for the *HERVH Xp22.2-AS* isoforms were amplified using the following primers (Table 6):

**Table 6:** Amplicon next-generation sequencing primers used in this study

*Gene name*	*Forward*	*Reverse*
HERVH Xp22.2 2-C	tcgtcggcagcgtcagatgtgtataagagacagCCGCTAAGCCGAGAAGATCTGGG	gtctcgtgggctcggagatgtgtataagagacagTCGAGAGGAAAGGGCTGTGTCC
HERVH Xp22.2 2–6	tcgtcggcagcgtcagatgtgtataagagacagTCAGTCTTCAGCCGCTAAGCCG	gtctcgtgggctcggagatgtgtataagagacagTGTGCCACATAAGGTGTCCACG
HERVH Xp22.2 2-altend	tcgtcggcagcgtcagatgtgtataagagacagAATCAGGCAGCGTCAGTCTT	gtctcgtgggctcggagatgtgtataagagacagAACAGCTGTGCCACATAAGG
HERVH Xp22.2 2-end	tcgtcggcagcgtcagatgtgtataagagacagTCTTCAGCCGCTAAGCCG	gtctcgtgggctcggagatgtgtataagagacagAAGACTGTGAGAACCCCAGG
HERVH Xp22.2 6-end	tcgtcggcagcgtcagatgtgtataagagacagTGGAATTAACAACGTGGACACC	gtctcgtgggctcggagatgtgtataagagacagTGAGAGGATGGTCTAGGGCT
HERVH Xp22.2 6-altend	tcgtcggcagcgtcagatgtgtataagagacagTGGAATTAACAACGTGGACACC	gtctcgtgggctcggagatgtgtataagagacagGCATGCTTCTCAGATACAGGT

Lower-case and upper-case characters denote the sequencing library adaptors and isoform-specific sequences, respectively. PCR reactions were cleaned-up using the AMPure XP beads (Beckman Coulter) and were sequenced on the Illumina MiSeq platform with a PE 250-bp run configuration on a Nano flowcell

### Immunofluorescence

HEK293 T cells transfected with pcDNA3.1-CHRNA5 or pcDNA3.1-CHRNA5[AluSz] were grown on 1.5-mm coverglass dishes (MatTek, Cat #P35G-1.5–20-C) that were fixed using 10% neutrally buffered formalin (Genta Medical) for 15 min. Non-specific staining of non-permeabilised cells was blocked with 1% bovine serum albumin (Sigma-Aldrich). Primary antibody incubation for influenza hemagglutinin (HA)-tag antibody (Santa Cruz Biotechnology, sc-7392) was performed overnight at 4 °C at a 1:100 dilution. Secondary antibody incubation using Goat Anti-Mouse IgG H&L AlexaFluor594 Antibody (Abcam, Cat #ab150116) was carried out the next day for 1 h at room temperature at a 1:1000 dilution. Nuclear staining was performed using Hoechst 33,342 (Thermo Fisher). Samples were imaged on the Zeiss Observer.Z1 (Carl Zeiss Meditec AG) using Micro-Manager 2.0 software.

### Retroviral transduction

Stably transduced cell lines were produced through viral infection and single cell sorting on GFP and mCherry using the MoFLO XDP cell sorter (BD Biosciences, Flow Cytometry Team, The Francis Crick Institute). Virus was generated using HEK293 T cells plated at a density of 1.5 × 10^6^ cells per 60 mm well incubated with a mixture of serum-free IMDM (Sigma-Aldrich, I3390), GeneJuice (VWR International Ltd., 70,967–4), and 5 μL of plasmid DNA. Plasmids used were vesicular stomatitis virus glycoprotein (VSVg) plasmid (pcVG-wt), pHIT60, and the open reading frames of the sequences of interest cloned into the pRV-IRES-GFP or pRV-IRES-mCherry vector (see Tables 2, 3, 4, 5 and 6). Cloning the open reading frames into the vector was carried out Genewiz LLC and was followed by sequencing to verify the plasmid structure. Virus-containing supernatant was collected and added to HEK293 T cells (plated at a density of 8.5 × 10^4^ cells per 35 mm well) along with polybrene (Sigma-Aldrich, TR-1003-G), and spun at 1200 RPM for 45 min. After 3 days, populations were single cell sorted on GFP, or mCherry expression using a BD FACSAria II (BD Biosciences) (Flow Cytometry STP, The Francis Crick Institute).

### Protein preparation for western blot

Cells were washed twice with phosphate-buffered saline (PBS) stored at 4 °C before being incubated on ice with radioimmunoprecipitation assay (RIPA, Sigma-Aldrich, R0278-50ML) buffer for 30 min to lyse the cells. The mixture was then spun at 14,000 RPM for 10 min at 4 °C. The protein concentration of the lysate was measured using the Pierce™ BCA protein assay kit (Thermo Scientific, 23,225). Stock solutions at a protein concentration of 500 μg/mL were made by mixing 100 μL of sample buffer (Laemmli 2 × concentrate, Sigma-Aldrich, S3401-10 VL), with 100 μg of protein lysate and RIPA buffer to a final volume of 200 μL. Stock solutions were heat denatured at 95 °C for 5 min before being frozen at − 20 °C.

### Western blot

Sample stock solutions were thawed on ice before being boiled at 95 °C for 5 min. Ten micrograms of protein per sample was loaded into a 4–20% Mini-PROTEAN® TGX™ precast polyacrylamide gel (Bio-Rad, 4,561,094) alongside a protein ladder (PageRuler Plus Prestained Protein Ladder, 10 to 250 kDa, ThermoFisher, 26,619). The gel electrophoresis was run in a Mini-PROTEAN® Tetra Vertical Electrophoresis Cell (Bio-Rad) filled with protein running buffer (Media Team, The Francis Crick Institute) at 180 V for 40 min. Samples were transferred to a 0.2-μm nitrocellulose membrane (Trans-Blot Turbo Mini 0.2 μm Nitrocellulose Transfer Pack, Bio-Rad, 1,704,158) using the Trans-Blot Turbo dry transfer system (Bio-Rad, 1,704,150) turbo setting for mini TGX gels before blocking with 5% skimmed milk (Marvel) in Tris-buffered saline with 0.5% Tween-20 (TBS-T, Media Team, The Francis Crick Institute) for 1 h. Membranes were stained overnight at 4 °C with the anti-FLAG antibody (Sigma-Aldrich, F1804) diluted at 1:1000 in 5% skimmed milk in TBS-T. Membranes were washed with TBS-T at room temperature before the horseradish peroxidase (HRP)-conjugated anti-mouse secondary antibody (Abcam, ab6728) was added, diluted at 1:1000 in 3% skimmed milk in TBS-T and incubated at room temperature for 1 h. Membranes were then washed in TBS-T and visualised by enhanced chemiluminescence using Clarity™ Western ECL Substrate (Bio-Rad, 1,705,060) on a ChemiDoc XRS + (Bio-Rad).

### Transwell migration assay

Cell migratory capacity was examined by transwell migration assay that uses chemotactic gradient to assess how cells migrate through a porous membrane. Cells were trypsinised and resuspended in serum-free media. They were then seeded into Millicell Hanging Cell Culture Insert (Millipore, PTEP24H48) at a density of 20 × 10^3^ for A498 and A549 clones, while the lower chamber contained fresh culture media with 30% FBS. The cells were allowed to migrate for at least 48 h. The inserts were washed with PBS and fixed with 10% neutrally buffered formalin (Genta Medical) for 10 min. Those cells that did not migrate were removed. The cells on the lower surface of the insert were washed with PBS again, stained with crystal violet solution (Sigma Aldrich, V5265). The cells on each insert were counted in 4 random fields under Zeiss Observer.Z1 (Carl Zeiss Meditec AG) using Micro-Manager 2.0 software.

### Cell growth assays

Growth and proliferation of A498 parental control (1E7), A498 *HERVE 6q15*^*−/−*^ (2D11) clone, A549 parental and canonical *CHRNA5* and *CHRNA5[AluSz]* expressing A549 clones was assessed by real-time quantitative live-cell imaging using the Incucyte Live-Cell Analysis System (Sartorius). Cells were seeded into 96-well plates 24 h prior measurement in Incucyte system at a density of 2000 and 1000 cells per well for A498 and A549, respectively. Image and confluency measurement were taken every 3 h for at least 72 h. Cell growth data for RNGTT-deficient cell lines in CCLE were downloaded from Dependency Map (DepMap) portal [35].

### Statistical analyses

Statistical comparisons were made using GraphPad Prism 10.3 (GraphPad Software), SigmaPlot 14.0, Qlucore Omics Explorer v3.9, or R (versions 3.6.1–4.0.0). Parametric comparisons of normally distributed values that satisfied the variance criteria were made by unpaired or paired Student’s *t*-tests or one-way analysis of variance (ANOVA) tests with Bonferroni correction for multiple comparisons. Data that did not pass the variance test were compared with non-parametric two-tailed Mann–Whitney rank sum tests (for unpaired comparisons) or Kruskal–Wallis test with Dunn’s multiple comparisons correction.

## Results

### Widespread potential for RTE-mediate disruption of adjacent gene function

**Fig. 1:**
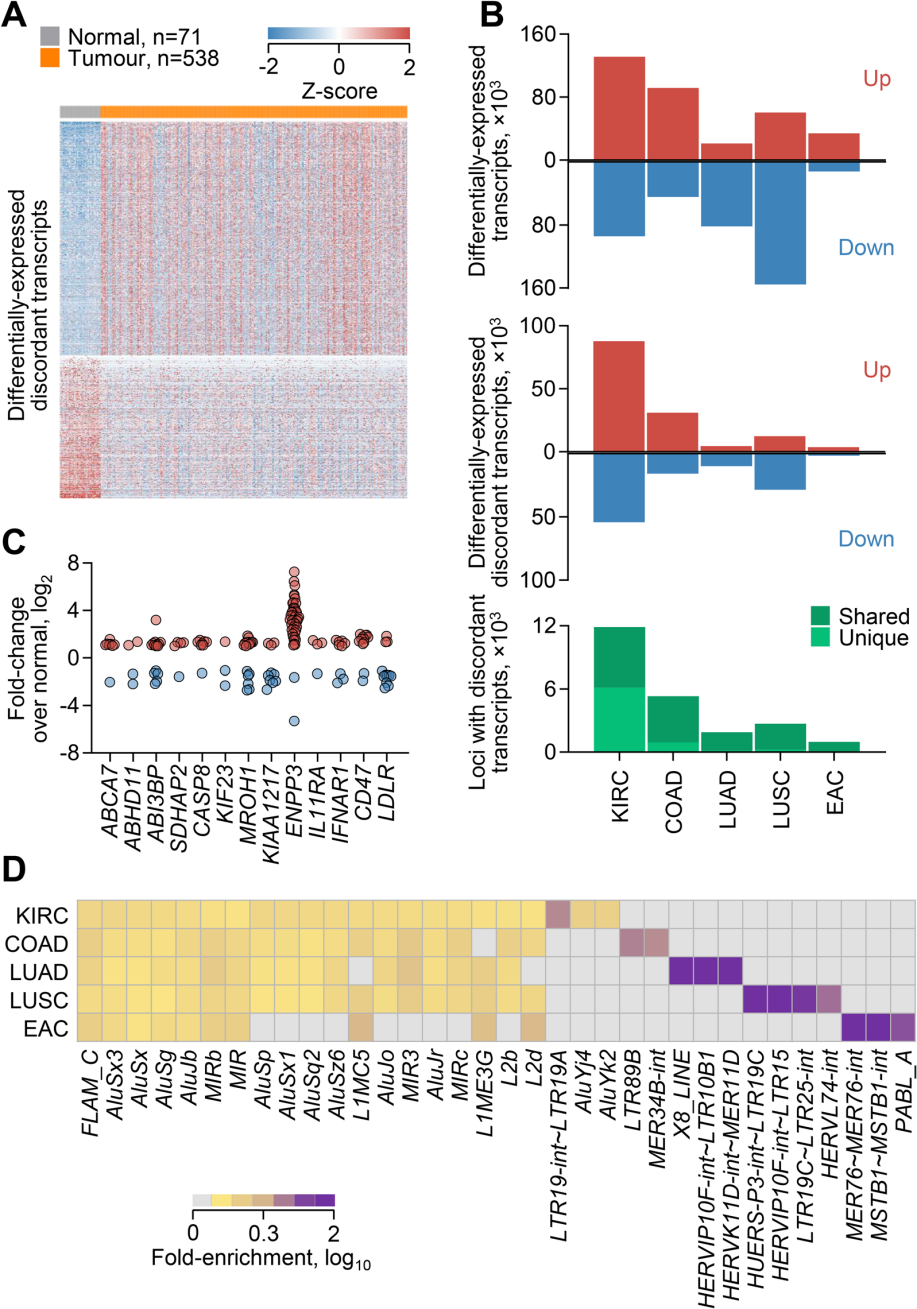
Widespread shifts in the balance of RNA isoform expression in cancer. **A** Heatmap of expression of 87,861 and 54,202 discordant transcripts that are upregulated and downregulated (≥ 1.5 fold-change, *p* ≤ 0.05, *q* ≤ 0.05), respectively, in TCGA KIRC samples, compared with normal kidney tissue, and overlap with loci producing transcripts in both categories. **B** Total number of differentially expressed transcripts (≥ 1.5 fold-change, *p* ≤ 0.05, *q* ≤ 0.05) (*top*), differentially expressed discordant transcripts (*middle*), and loci producing discordant transcripts (*bottom*) between the indicated cancer types and their respective normal tissue (KIRC *n* = 538, normal kidney *n* = 71; COAD *n* = 239, normal colon *n* = 39; LUAD *n* = 419, normal lung *n* = 24; LUSC *n* = 362, normal lung *n* = 24; EAC *n* = 78, normal oesophagus *n* = 9). **C** Fold-change of individual discordant transcripts (symbols) overlapping with the indicated loci in KIRC samples. **D** Fold-enrichment for the indicated RTE subgroups in discordant transcripts, compared with the entire transcriptome. All indicated RTE subgroups were significantly enriched (*p* ≤ 0.05)

To identify cases where aberrant transcriptional inclusion of RTEs may affect local gene function, we searched for gene loci that exhibit a specific switch in RNA isoform expression. To this end, we used a cancer transcriptome assembly that captures diverse RTE-overlapping transcripts and quantified transcript abundance using Salmon, which uses a probabilistic model for assignment of reads aligning to multiple transcript isoforms [10]. We subsequently selected transcripts that were either upregulated or downregulated in a given cancer type compared with its respective normal tissue (Fig. 1A–C). Intersection of the two lists identified transcripts that, despite exhibiting contrasting transcriptional behaviour (referred to here as discordant transcripts), were transcribed from the same locus (Fig. 1A, B). For example, from a total of 225,544 transcripts differentially expressed between KIRC and normal kidney tissue, 62.9% were from loci that produced transcriptionally discordant RNA isoforms (Fig. 1A–C), suggesting extensive changes in RNA isoform balance in this cancer type. Similar results were also obtained from analysis of COAD, LUAD, LUSC and EAC, although the proportion of discordant transcripts was lower (11.3–34.3%) in these cancer types (Fig. 1C). Moreover, nearly half of the loci producing transcriptionally discordant RNA isoforms in KIRC were also found in at least one other cancer type, and this fraction was much higher (82.5–92.2%) for the remaining types (Fig. 1C). Compared with the entire previously assembled transcriptome, *Alu*, *MIR* and *L1* elements were particularly enriched in discordant transcripts from all types of cancer analysed, whereas LTR elements displayed a cancer type-specific pattern of enrichment (Fig. 1D). These findings suggested extensive imbalances in RNA isoform expression in cancer through increased RTE utilisation and indicated common underlying mechanisms operating in multiple cancer types. Given the potential of alternative isoforms generated by RTE co-option to affect gene function or create new function, we next investigated specific effects on nearby or encompassing genes in detail.

### Downregulation of *RNGTT* transcription by an intronic *HERVE* integration

**Fig. 2:**
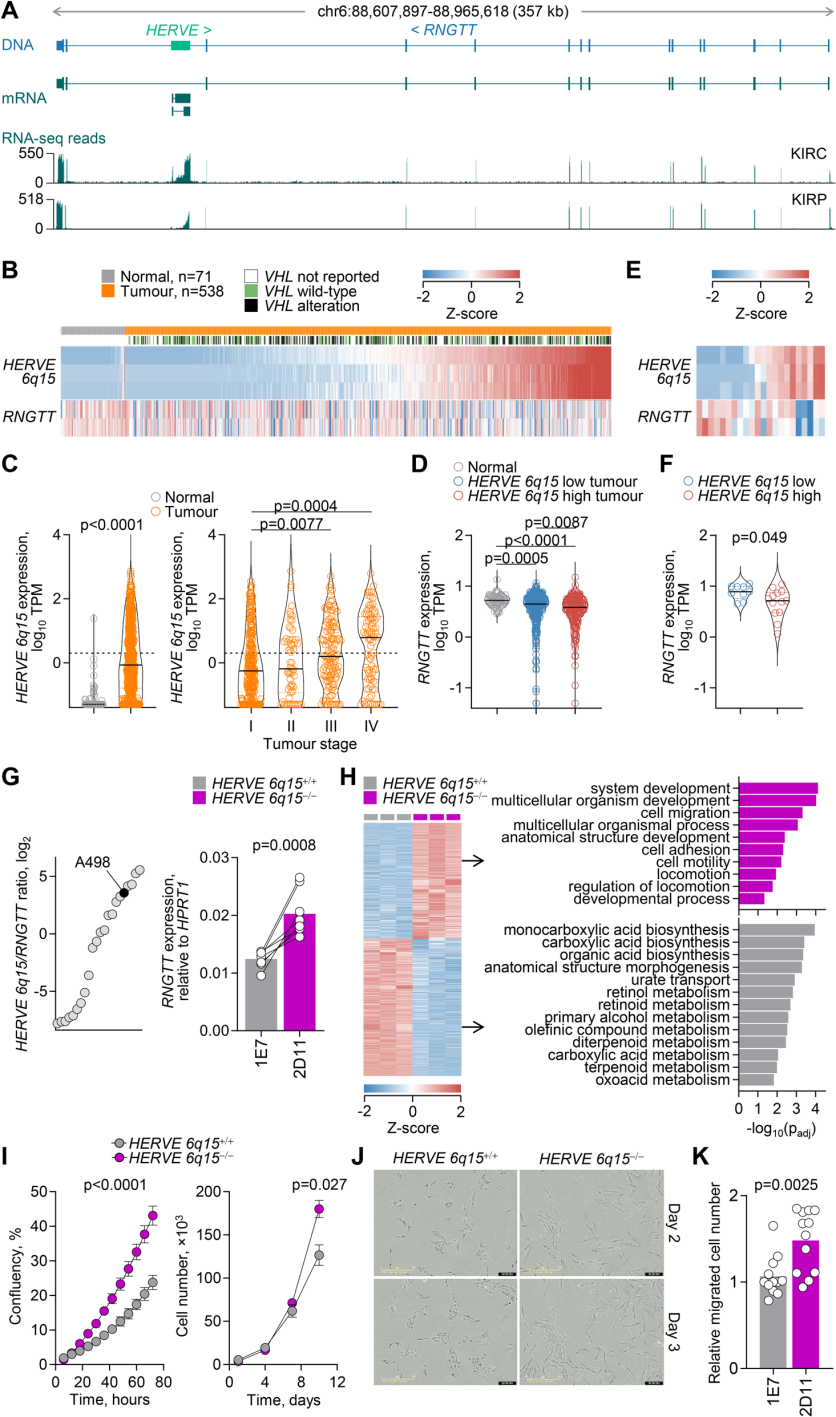
Effect of *HERVE* activation on *RNGTT* transcription. **A** Gene structure and integrated *HERVE* provirus, GENCODE-annotated and assembled transcripts, and RNA-seq traces of 24 combined KIRC and kidney renal papillary cell carcinoma (KIRP) samples at the *RNGTT* locus. **B** Expression of transcripts overlapping *HERVE 6q15* or the canonical *RNGTT* in normal kidney tissue and KIRC samples, ordered according to *HERVE 6q15* expression. **C***HERVE 6q15* expression in TPM in the same samples as in **B** (*p* value calculated with Mann–Whitney test), and according to tumour stage (I *n* = 271, II *n* = 59, III *n* = 123, IV *n* = 82; *p* values calculated with Kruskal–Wallis test with Dunn’s multiple comparisons correction). **D***RNGTT* expression (TPM) in normal kidney tissue (*n* = 71) and in KIRC samples with low (*n* = 322) and high (*n* = 216) *HERVE 6q15* expression (*p* values calculated with Kruskal–Wallis test with Dunn’s multiple comparisons correction). **E** Expression of transcripts overlapping *HERVE 6q15* or the canonical *RNGTT* in renal cell carcinoma cell lines in CCLE, in columns ordered according to *HERVE 6q15* expression. **F***RNGTT* expression (TPM) in renal cell carcinoma cell lines with low (*n* = 9) and high (*n* = 13) *HERVE 6q15* expression (*p* value calculated with two-tailed Student’s *t*-test). **G** Ratio of *HERVE 6q15* to *RNGTT* expression in renal cell carcinoma cell lines in CCLE (*left*), and *RNGTT* expression (assessed by RT-PCR and plotted relatively to *HPRT1* expression) in A498 cells with (clone 1E7, *HERVE 6q15*^+/+^) or without (clone 2D11, *HERVE 6q15*^*−*/*−*^) the *HERVE 6q15* provirus (*right*). Symbols represent replicates of 4 independent experiments, connected with lines (*p* value calculated with two-tailed paired Student’s *t*-test). **H** Heatmap of differential gene expression (≥ twofold-change, *p* ≤ 0.05, *q* ≤ 0.05) of between *HERVE 6q15*^+/+^ and *HERVE 6q15*^*−*/*−*^ A498 cells (*left*). Columns represent independent replicates. Gene ontology (GO) functional annotation of the differentially expressed genes (*right*) (*p* values calculated with g:Profiler using hypergeometric distribution tests and adjusted for multiple hypothesis testing using the g:SCS (set counts and sizes) algorithm, integral to the g:Profiler server [33]. **I** Mean confluency (± SD, *n* = 6 from 1 experiment) (*left*) and mean cell number (± SEM, *n* = 3 from 1 experiment) (*right*) of *HERVE 6q15*^+/+^ and *HERVE 6q15*^*−*/*−*^ A498 cell cultures over time (*p* values calculated by two-tailed Student’s *t*-test of the area under the curve (AUC) values and by two-tailed Student’s *t*-test for day 10, respectively). **J** Representative images of *HERVE 6q15*^+/+^ and *HERVE 6q15*^*−*/*−*^ A498 cell morphology 2 or 3 days after plating. **K** In vitro migration of *HERVE 6q15*^+/+^ (1E7) and *HERVE 6q15*^*−*/^.^*−*^ (2D11) A498 cells. Symbols represent independent measurements (*n* = 12, 4 fields of view from 3 independent experiments; *p* value calculated with two-tailed Student’s *t*-test)

An example of a locus producing discordant transcripts in KIRC was *RNGTT* (RNA guanylyltransferase and 5′-phosphatase), encoding the mRNA capping enzyme with RNA 5′-triphosphate monophosphatase and guanylyltransferase activities. In its penultimate intron, *RNGTT* harbours a *HERVE* provirus, integrated in reverse orientation relative to *RNGTT* (Fig. 2A). This *HERVE* provirus, referred to here as *HERVE 6q15*, is known to be highly expressed in KIRC and to encode immunogenic retroviral proteins, which contribute to tumour immune control [36–39]. However, the consequences of its transcriptional induction on *RNGTT* have not been previously considered. We found that transcription of *RNGTT* and the intronic *HERVE* provirus exhibited an inverse pattern in KIRC (Fig. 2B–D). *HERVE 6q15* was not expressed in normal kidney tissue but was highly induced in ~ 50% of KIRC cases, with significantly higher expression in later stages of the disease (Fig. 2B, C). In contrast, *RNGTT* expression was significantly reduced in KIRC, compared with normal kidney tissue, and this reduction was more pronounced in cases with higher *HERVE 6q15* expression (Fig. 2B, D), suggesting that transcriptional activation of the *HERVE* integration negatively impacted *RNGTT* expression. A similar negative correlation between *RNGTT* and *HERVE 6q15* transcription was also observed in RNA-seq data from individual renal cell carcinoma cell lines obtained from CCLE (Fig. 1E, F), where possible confounding effects of non-tumour cells in tumour samples can be excluded.

In agreement with prior reports [37], *HERVE 6q15* transcription in KIRC was directly correlated with the degree of hypoxia (Additional file 1: Fig. S1 A, B), although the strength of this correlation was likely affected by the reliability of current hypoxia scores to reflect the pseudohypoxic state of this cancer type [30]. However, a direct effect of hypoxia on *HERVE 6q15* transcription was evident by analysis of RNA-seq data from renal cell carcinoma RCC4 cells with restored expression of the VHL tumour suppressor [20] (GSE120887) (Additional file 1: Fig. S1 C, D). Hypoxia significantly increased *HERVE 6q15* expression in VHL-expressing RCC4 cells, but did not affect levels of *RNGTT* transcription, which were already very low and substantially lower than those of *HERVE 6q15* in these cells (Additional file 1: Fig. S1 C, D).

The enzymatic activities encoded by *RNGTT* play an indispensable role in RNA capping, by catalysing the first step of a complex series of reactions leading to the addition of the methyl-7-guanosine cap on the 5′ ends of nascent RNAs [40]. In turn, RNA capping affects all subsequent aspects of RNA processing and function, including translation potential [40]. Accordingly, *RNGTT* is a common essential gene, required for growth of virtually all cancer cell lines (Additional file 1: Fig. S2), and its direct pro-tumour activity is associated with worse prognosis on most cancer types [40–42].

Given that high levels of *HERVE 6q15* transcription in most renal cell carcinoma cell lines may have reduced *RNGTT* transcription to levels that cannot be further reduced without compromising cell viability (Additional file 1: Fig. S2), we investigated a direct effect of *HERVE 6q15* on *RNGTT*, with the reverse experiment. We selected renal cell carcinoma A498 cells, which express a high *HERVE 6q15* to *RNGTT* ratio (Fig. 1G), and used CRISPR/Cas9 editing to remove the *HERVE* provirus, together with two immediately adjacent *L1* integrations (Additional file 1: Fig. S3). Assessed by RT-qPCR and compared with the *HERVE 6q15*^+*/*+^ clone (1E7), expression of *RNGTT* was upregulated by ~ 63% in the *HERVE 6q15*^*−/−*^ clone (2D11) (Fig. 1G). *RNGTT* upregulation following *HERVE 6q15* deletion in A498 cells was accompanied by extensive transcriptional changes, with upregulation of genes involved in cell adhesion and migration, and downregulation of genes involved in metabolic processes (Fig. 1H). Consistent with their transcriptional profile and the pro-tumour activities of *RNGTT*, *HERVE 6q15*^*−/−*^ 2D11 cells exhibited increased in vitro growth, altered morphology and increased migration (Fig. 1I–K). Lastly, supporting opposing transcriptional profiles, *RNGTT* and *HERVE 6q15* levels also showed the opposite correlation with KIRC survival, which was however an indirect result of *HERVE 6q15* association with later stages of the disease (Additional file 1: Fig. S4). Together, these results support a model of *HERVE 6q15* transcriptional induction during KIRC progression, which reduces, but does not abolish pro-tumour *RNGTT* expression.

Regulation of cadherin 4 by *THE1 A*-driven antisense transcription.

**Fig. 3:**
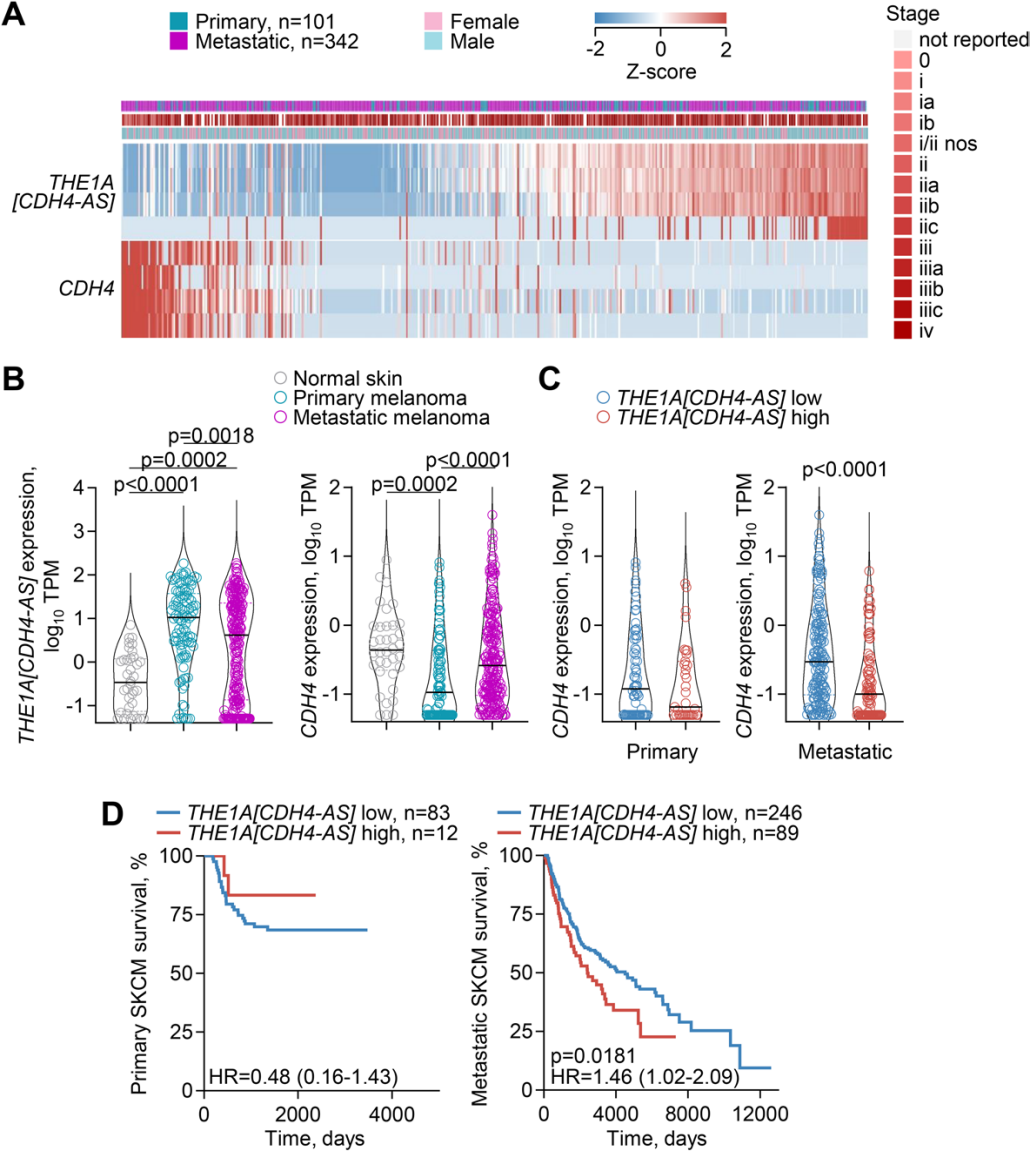
Effect of *THE1 A* activation on *CDH4* transcription. **A** Expression of transcripts overlapping *THE1 A[CDH4-AS]* or the canonical *CDH4* in primary and metastatic SKCM samples. **B ***THE1 A[CDH4-AS]* and canonical *CDH4* expression (TPM) in normal skin tissue (*n* = 36) and in primary (*n* = 101) and metastatic (*n* = 224) SKCM samples (*p* values calculated with Kruskal–Wallis tests with Dunn’s multiple comparisons correction). **C ***CDH4* expression (TPM) in primary and metastatic SKCM samples with low (*n* = 66 and *n* = 161, respectively) and high (*n* = 35 and *n* = 89, respectively) *THE1 A[CDH4-AS]* expression (*p* value calculated with Mann–Whitney test). **D** Overall survival of primary and metastatic SKCM patients, stratified by *THE1 A[CDH4-AS]* expression (*p* values calculated with log-rank tests)

We have previously identified an antisense transcript, initiated by a *THE1 A* retroelement and referred to as *THE1 A[CDH4-AS]*, which is spanning two introns of the *CDH4* gene (encoding cadherin 4) and expressed specifically in cutaneous and uveal melanomas [10]. To examine a possible effect of transcriptional activation of the intronic *THE1 A* element on *CDH4* transcription, we correlated the two in RNA-seq data from TCGA SKCM samples (Fig. 3). This analysis revealed a pattern of mutual exclusivity between *THE1 A[CDH4-AS]* and *CDH4* transcription in both primary and metastatic SKCM, irrespective of disease stage (Fig. 3A). However, compared with normal skin samples, the transcriptional activation of the *THE1 A* element and concomitant downregulation of *CDH4* transcription were considerably more pronounced in primary than in metastatic disease (Fig. 3B, C). Indeed, whereas significantly reduced in primary melanoma, overall *CDH4* transcription remained high in metastatic melanoma, particularly in samples with low *THE1 A[CDH4-AS]* transcription (Fig. 3B, C).

Cadherin 4, also known as R-cadherin (retinal), is a member of the calcium-dependent adhesion molecule superfamily, important in forming adherens junctions and in organ development [43, 44]. Cadherin-regulated cellular adhesion and detachment also determines migration and invasiveness of tumour cells and, consequently, the ability of several cancer types to metastasise [45, 46]. In the skin, E-cadherin (epidermal) and P-cadherin (placental) mediate heterotypic adhesion of neural crest-derived melanocytes with the surrounding epithelial cells [47]. Loss of E-cadherin and P-cadherin, and gain of N-cadherin (neuronal), known as cadherin switching [46], facilitates melanoma cell metastasis and is associated with worse prognosis of SKCM [48]. While its role in melanoma is less well studied, R-cadherin mediates adhesion with N-cadherin [43] and its overexpression in epidermoid carcinoma A431 cells causes the loss of E-cadherin and P-cadherin, through competition for the intracellular adaptor proteins catenins [49]. An involvement of R-cadherin in cadherin switching is further supported by reports of an essential role in tumorigenesis and metastasis in human osteosarcoma [50] and in a murine model of glioma [51], and of a tumour suppressive function in human colorectal and gastric cancers [52].

Consistent with a role for *CDH4* in the metastatic process suggested by findings in other cancer types, we found that transcriptional co-option of *THE1 A[CDH4-AS]* specifically in SCKM differentiates primary and metastatic disease and is differentially associated with survival in the two subtypes (Fig. 3D). This association indicated that *THE1 A[CDH4-AS]*-mediated suppression of *CDH4* in primary melanomas restrains their metastatic potential, whereas the failure to establish or the loss of such transcriptional effect facilitates metastasis.

*HERVH*-driven downregulation of endosomal single-stranded RNA sensors TLR7 and TLR8.

**Fig. 4:**
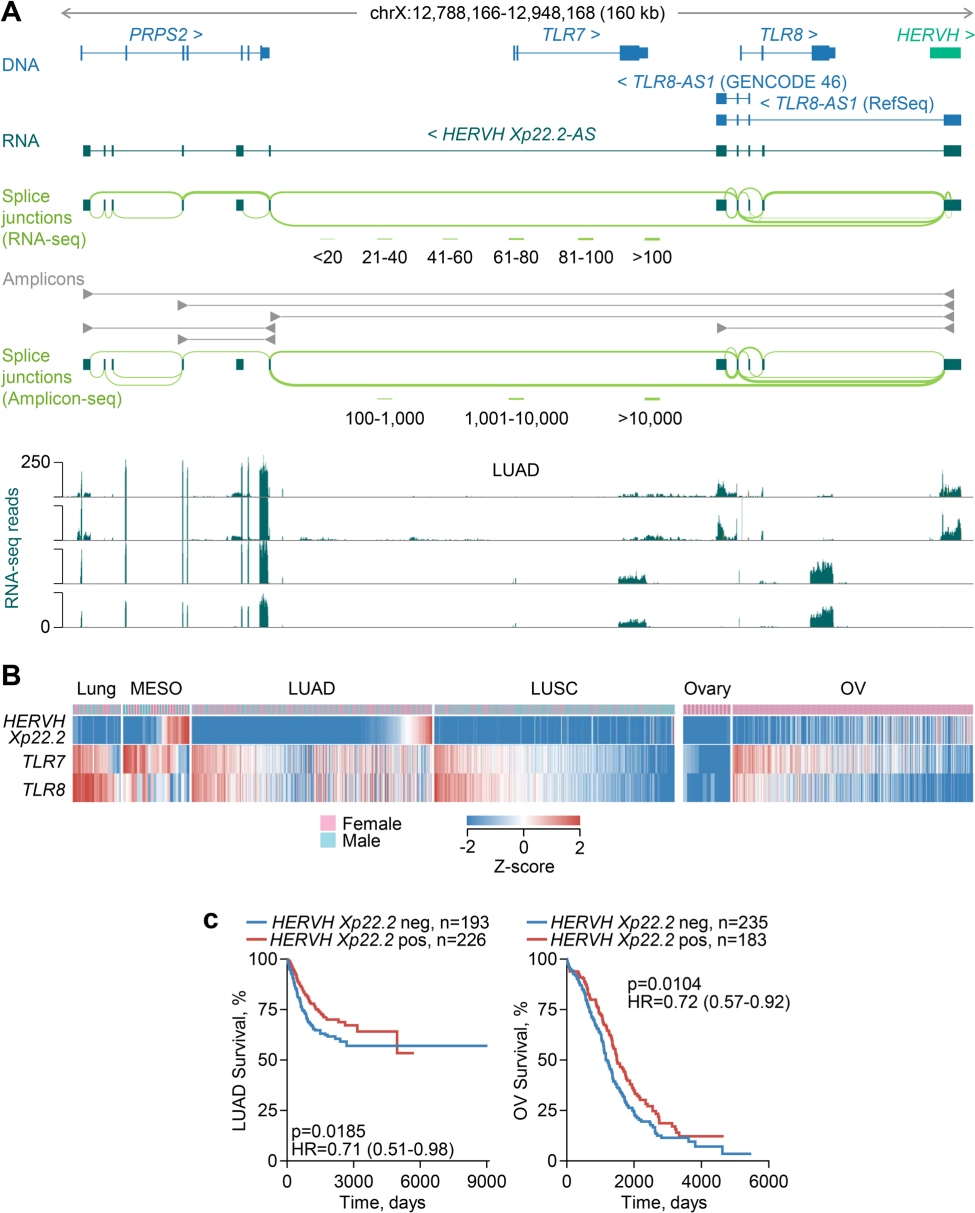
Effect of *HERVH* activation on *TLR7* and *TLR8* transcription. **A** Gene structure and integrated *HERVH* provirus at the *PRPS2-TLR7-TLR8* locus, GENCODE- and RefSeq-annotated and assembled transcripts, splice junction analysis of LUAD RNA-seq data, amplicons used for transcript validation, splice junction analysis of amplicon sequencing, and RNA-seq traces of LUAD samples with (*top two*) or without (*bottom two*) *HERVH* transcriptional activation. **B** Expression of transcripts overlapping *HERVH Xp22.2* or the canonical *TLR7* or *TLR8* in normal lung (*n* = 36) and ovary (*n* = 12) tissue, and in MESO (*n* = 24), LUAD (*n* = 419), LUSC (*n* = 362) and OV (*n* = 419) samples. **C** Overall survival of LUAD (*left*) and OV (*right*) patients, stratified by *HERVH Xp22.2* expression (*p* values calculated with log-rank tests)

The *TLR7* and *TLR8* paralogue genes, two members of the Toll-like receptor (TLR) family, are arranged in tandem on chromosome Xp22.2, followed by a *HERVH* provirus, referred to here as *HERVH Xp22.2* (Fig. 4A). Our assembly identified a number of antisense transcripts, initiated by the bidirectional promoter activity of the *HERVH Xp22.2* provirus, using a total of 10 alternative exons spread throughout the locus and extending to the upstream gene *PRPS2* (phosphoribosyl pyrophosphate synthetase 2) (Fig. 4A). Some of the assembled transcripts partially matched the annotated *TLR8-AS1* transcripts in GENCODE 46 (ENST00000451564) and RefSeq (NR_030727), which appeared incomplete (Fig. 4A). Splice junction analysis of RNA-seq data from TCGA LUAD samples revealed two main groups of *HERVH Xp22.2*-initated antisense transcripts (collectively referred to as *HERVH Xp22.2-AS*), one terminating between the *TLR7* and *TLR8* loci and one terminating within the *PRPS2* locus (Fig. 4A). To validate the complex splicing pattern, we amplified the corresponding cDNAs from key splicing isoforms expressed in lung adenocarcinoma HCC4006 cells. Deep-sequencing of the amplicons confirmed the alternative use of middle and terminal exons, as well as the balance of shorter and longer *HERVH Xp22.2-AS* isoforms (Fig. 4A).

Across several cancer types and respective normal tissues, *HERVH Xp22.2-AS* was transcriptionally activated in a substantial proportion of samples from OV, MESO and testicular germ cell tumours (TGCT), and a smaller proportion of samples from other cancers, including LUAD and LUSC, but remained inactive in normal tissues, with the possible exception of a small number of Epstein-Barr virus (EBV)-transformed B cell lines (Additional file 1: Fig. S5 A). Notably, *HERVH Xp22.2-AS* transcription was strongly anti-correlated with *TLR7* and *TLR8* transcription in cancers where it was expressed (Fig. 4A, B; Additional file 1: Fig. S5B). Indeed, whereas normal lung tissue expressed *TLR7* and *TLR8* highly and proportionally, without detectable *HERVH Xp22.2-AS* expression, ~ 13% of LUAD samples expressed high levels of *HERVH Xp22.2-AS*, but not of *TLR7* and *TLR8* (Additional file 1: Fig. S5B), indicating an inhibitory effect of *HERVH Xp22.2-AS* transcriptional activation on sense transcription. This effect appeared to extend also to the *PRPS2* locus, which was expressed only in LUAD samples, but not in normal lung tissue, and exhibited a significant anti-correlation with *HERVH Xp22.2-AS* expression (Additional file 1: Fig. S5B). Similar results were obtained also in LUSC, as well as MESO and OV, where *HERVH Xp22.2-AS* was highly expressed and *TLR7* and *TLR8* were downregulated in nearly half of the cases (Fig. 4B).

TLR7 and TLR8 are endosomal sensors of single-stranded RNA [53] that play essential roles in the defence against viral infection and in the induction of B cell systemic autoimmunity [54]. Recent studies have indicated an essential, yet dual role for TLR7 and TLR8 also in cancer progression and immune control [55, 56]. Whereas their ligation in immune cells may enhance anti-tumour activity, TLR7 and TLR8 are also expressed in tumour cells where they mediate a pro-tumour effect [55, 56]. Indeed, tumour cell-intrinsic expression of TLR7 and TLR8 promotes their growth and survival in vitro [57, 58] and is associated with poor clinical outcome in non-small cell lung carcinomas (NSCLC) [59–61]. This pro-tumour effect of TLR7 is further supported by studies in animal models [59].

A pro-tumour effect of tumour cell-intrinsic TLR7 and TLR8 expression would predict that their downregulation by *HERVH Xp22.2-AS* transcriptional activation has an anti-tumour effect. Although survival analyses of *TLR7* and *TLR8* expression are confounded by their expression both in tumour cells and in immune cells, the strict tumour specificity of *HERVH Xp22.2-AS* expression reflects tumour cell-intrinsic transcriptional states. Indeed, we found that higher *HERVH Xp22.2-AS* expression in tumour samples is significantly associated with better prognosis in both LUAD and OV (Fig. 4B), consistent with a protective effect of *HERVH Xp22.2-AS* transcriptional activation.

### Downregulation of *APOBEC3B* expression by *MER11 C* element co-option

**Fig. 5:**
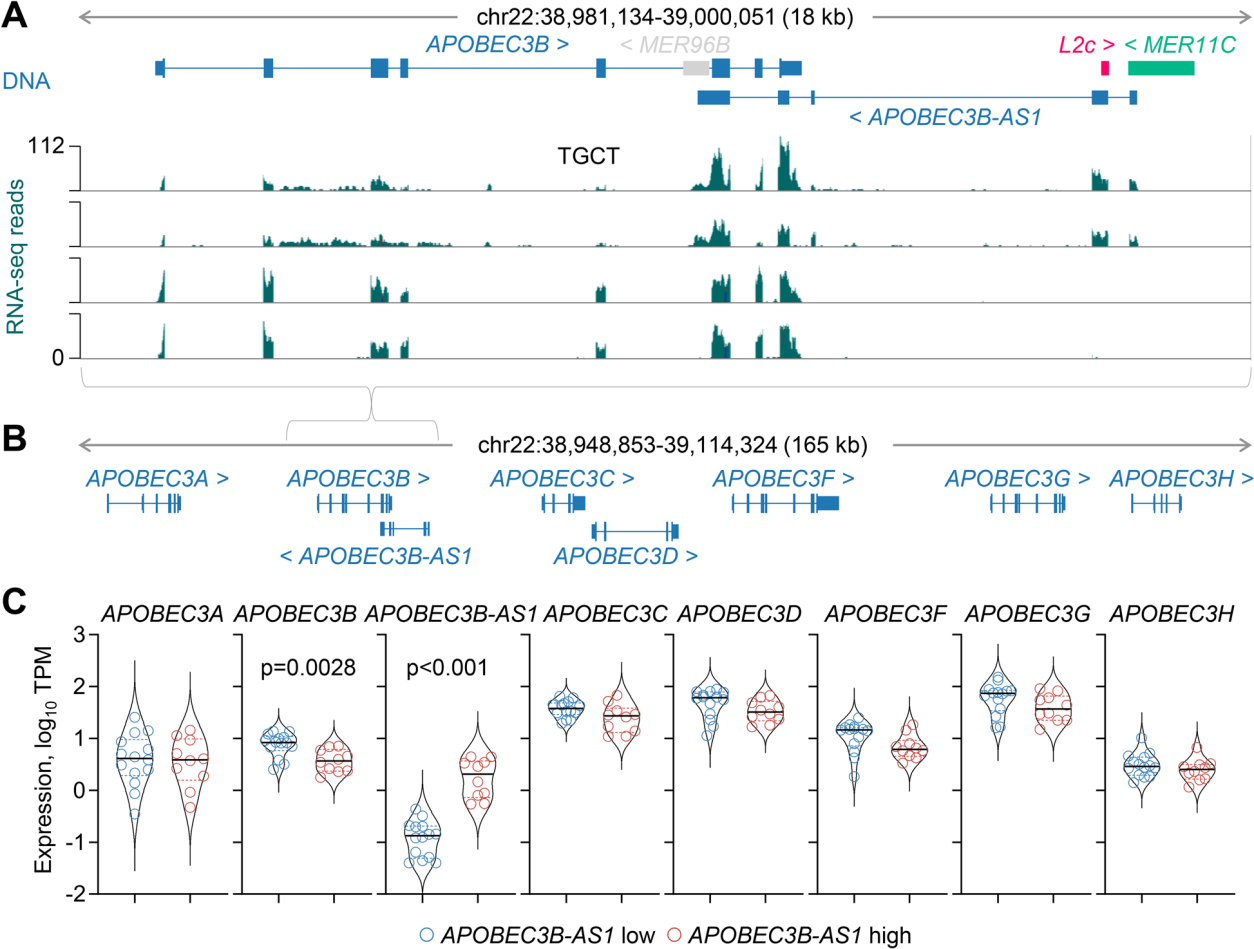
Effect of *MER11 C* activation on *APOBEC3B* transcription. **A** Gene structure and integrated *MER11 C*, *L2c* and *MER96B* RTEs at the *APOBEC3B* locus, and RNA-seq traces of TGCT samples with (*top two*) or without (*bottom two*) *MER11 C* transcriptional activation. **B** Gene structure at the extended *APOBEC3* gene cluster. **C** Expression (TPM) of *APOBEC3B-AS1* and the indicated *APOBEC3* genes in TGCT samples with low (*n* = 14) and high (*n* = 10) *APOBEC3B-AS1* expression (*p* values calculated with Mann–Whitney test and Student’s *t*-test for *APOBEC3B-AS1* and *APOBEC3B*, respectively)

Similar to *TLR7* and *TLR8* paralogues, members of the apolipoprotein B editing complex 3 (APOBEC3) family of enzymes are encoded by genes arranged in a cluster on chromosome 22 (Fig. 5A, B). They catalyse cytidine deamination in DNA or RNA substrates, which can potently inhibit virus and RTE replication, but can also drive genomic diversity and instability in cancer [62, 63]. Current evidence implicates APOBEC3 A and APOBEC3B as the two enzymes primarily responsible for the mutational signatures in human cancers and indicates a role for APOBEC3B in the regulation of APOBEC3 A [64]. Expression of human APOBEC3B in mice enhances their susceptibility to tumours and also causes male infertility [65], supporting a pro-tumour role, as well as a detrimental effect on the genetic integrity of the male germline.

In this locus, we have identified a transcript matching annotated transcript *APOBEC3B-AS1* (ENST00000513758) and transcribed in the reverse orientation in relation to the *APOBEC3* genes (Fig. 5A, B). This transcript was initiated by a *MER11 C* element integrated between the *APOBEC3B* and *APOBEC3 C* genes and extended over the first 3 *APOBEC3B* exons (Fig. 5A, B). High *APOBEC3B-AS1* expression was highly specific to TGCT samples, with very low expression in other cancer types or normal tissues (Additional file 1: Fig. S6). Importantly, transcriptional activation of the *MER11 C* element in TGCT samples was accompanied by reduction specifically in transcription of the overlapping *APOBEC3B* gene, whereas transcription of all other *APOBEC3* genes in this cluster remained unaffected (Fig. 5C).

### Reduction of ENPP3 potential by a switch to non-functional isoforms

**Fig. 6:**
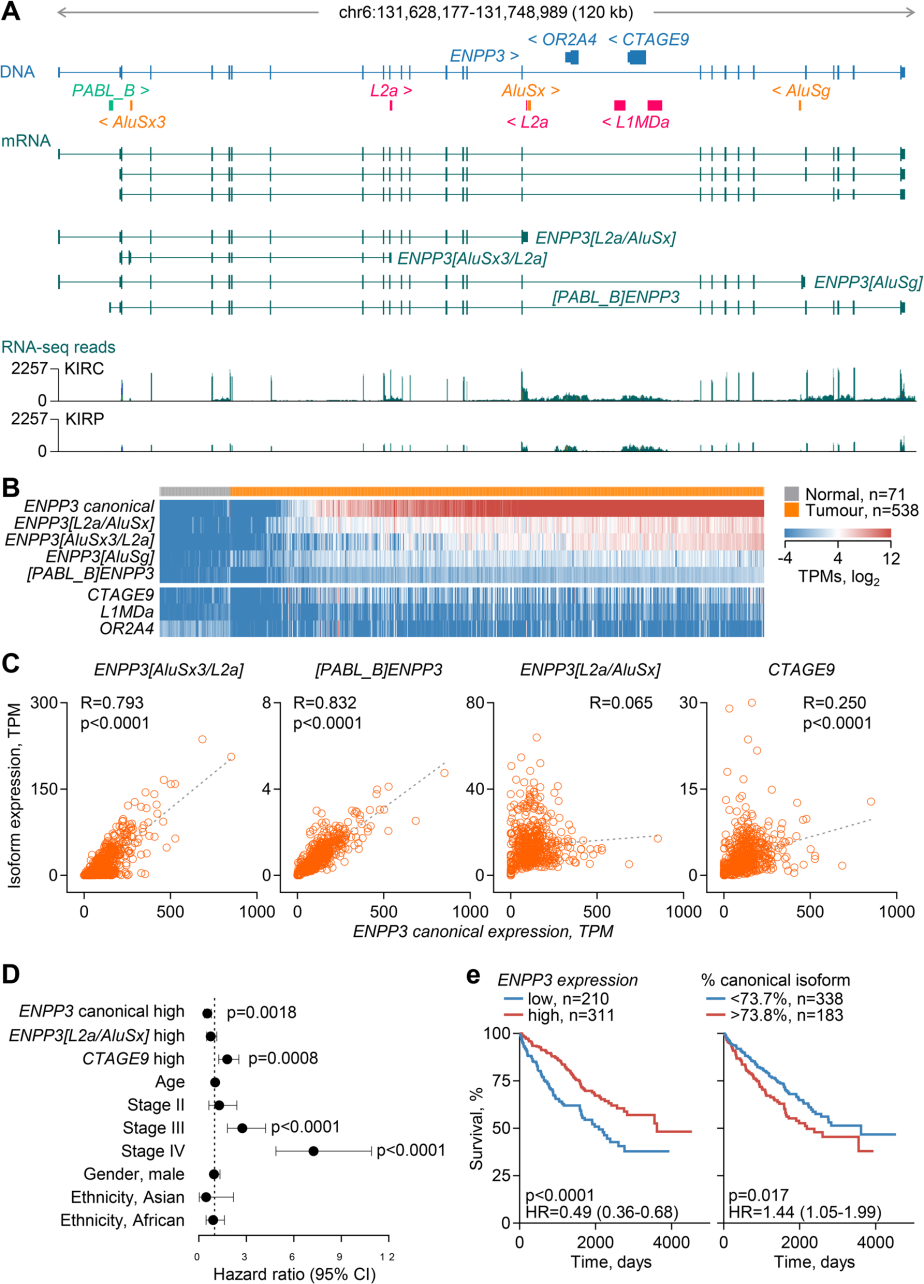
Effect of local RTEs on *ENPP3* functional and non-functional isoform balance. **A** Gene structure and exonised RTEs, GENCODE-annotated and assembled transcripts, and RNA-seq traces of 24 combined KIRC and KIRP samples at the *ENPP3* locus. **B** Expression of transcripts overlapping the indicated *ENNP3* isoforms or overlapping genes and RTEs in normal kidney tissue and KIRC samples, ordered according to canonical *ENPP3* expression. **C** Correlation of *ENPP3* isoform and *CTAGE9* expression (TPM) in KIRC samples (*n* = 538) (*p* values calculated with linear regression). *CTAGE9* expression is capped at 30 TPM. **D** Overall survival hazard ratios (HRs) for the indicated variables in KIRC patients (*ENPP3* canonical high *n* = 311, reference low *n* = 210; *ENPP3[L2a/AluSx]* high *n* = 214, reference low *n* = 307; *CTAGE9* high *n* = 174, reference low *n* = 347; Age *n* = 521; Stage II *n* = 56, III *n* = 123, IV *n* = 82, reference I *n* = 257; Gender, male *n* = 338, reference female *n* = 183; Ethnicity, Asian *n* = 8, African *n* = 55, reference White *n* = 450). Error bars represent 95% CIs (*p* values calculated with Cox proportional hazards regression). **E** Overall survival of KIRC patients, stratified by *ENPP3* expression (*left*) or the fraction of the canonical *ENPP3* isoform in total *ENPP3* expression (*right*) (*p* values calculated with log-rank tests)

The *ENPP3* (ectonucleotide pyrophosphatase/phosphodiesterase 3) locus produced multiple discordant transcripts, the majority of which were highly upregulated (Fig. 1B). In addition to *ENPP3*, the locus also contained two other annotated genes, *CTAGE9* and *OR2 A4*, both located in intron 16 of *ENPP3* and transcribed in the reverse orientation (Fig. 6A). Inspection of the locus identified several novel isoforms created by transcriptional inclusion of RTEs (Fig. 6A). These included a transcript using *L2a* and *AluSx* elements (*ENPP3[L2a/AluSx]*) also in intron 16 as alternative terminal exon and polyadenylation site (Fig. 6A). They also included a transcript using *AluSx3* and *L2a* elements as alternative second and terminal exons, respectively (*ENPP3[AluSx3/L2a]*), partially matching annotated GENCODE 46 transcript ENST00000427707 (Fig. 6A). Two additional transcripts were created by the use of a *PABL_B* element as alternative promoter (*[PABL_B]ENPP3*) or an *AluSg* element as an alternative terminal exon (*ENPP3[AluSg]*) (Fig. 6A). Compared with normal kidney tissue, expression of *ENPP3* was found significantly elevated in KIRC samples, in agreement with prior reports [66, 67], as was expression of alternative *ENPP3* isoforms, as well as of *CTAGE9* and an *L1MDa* integration straddling *CTAGE9*, whereas expression of *OR2 A4* was similar (Fig. 6B; Additional file 1: Fig. S7 A). Alternative *ENPP3* isoform expression accounted for a substantial proportion (~ 32%) of total *ENPP3* transcription, with stable balance through progressive stages of the disease, and was primarily driven by the *ENPP3[L2a/AluSx]* and *ENPP3[AluSx3/L2a]* transcripts (Fig. 6B; Additional file 1: Fig. S7B). Notably, whereas other *ENPP3* isoforms were expressed proportionally with the canonical isoform, expression of *ENPP3[L2a/AluSx]* and *CTAGE9* did not follow this pattern and appeared to be independently regulated (Fig. 6C).

ENPP3, also known as CD203c, is a type II transmembrane protein that catalyses the hydrolysis of extracellular nucleotides [68]. It was originally identified as a basophil and mast cell activation marker, regulating allergic inflammation by hydrolysing extracellular ATP [68, 69]. More recently, ENPP3 has also been implicated in the regulation of extracellular levels of cGAMP (2′3′-cyclic guanosine monophosphate), a second messenger for the activation of the STING (stimulator of interferon genes) pathway and the production of type I IFNs in viral infection and cancer [70]. Supporting a pro-tumour role, loss of ENPP3 cGAMP hydrolase activity in mice renders them more resistant to primary tumour growth and metastasis [70]. In addition to regulating the tumour immune environment, cell-intrinsic expression of *ENPP3* has been reported to promote cell migration [71] and to be essential for the growth of renal cell carcinoma cell lines [67], further supporting a pro-tumour function. We, therefore, considered the potential activity of the alternative ENPP3 isoforms.

Exonisation of the *AluSx3* element after the first coding exon of in the *ENPP3[AluSx3/L2a]* isoform creates a premature termination after codon 53, producing a severely truncated product (UniProt ID: E7ETI7), unlikely to retain any function. The *ENPP3[L2a/AluSx]* isoform has the potential to produce a larger protein, retaining the transmembrane helix and most of the phosphodiesterase domain, but missing the nuclease domain (Additional file 1: Fig. S8 A), which could exert altered enzymatic activities. However, in contrast to the canonical isoform, which was readily detectable upon overexpression in HEK293 T cells, the *ENPP3[L2a/AluSx]* isoform failed to produce a product of the expected or higher mass, indicative of protein instability (Additional file 1: Fig. S8B). These results suggested that alternative *ENPP3* isoforms are non-functional and their production is, therefore, at the expense of the canonical, thereby compromising the maximum capacity of the locus to produce the ENPP3 enzymatic activity. In turn, the reduction in ENPP3 potential could impede tumour progression. In multivariate analyses, overall *ENPP3* transcription was associated with favourable outcome in KIRC, as previously reported [66, 67], whereas *CTAGE9*, which encodes the cutaneous T cell lymphoma-associated antigen 9, showed the inverse association (Fig. 6D). Pertinently, a low proportion of canonical *ENPP3* among *ENPP3* isoforms was significantly associated with better survival in KIRC (Fig. 6E), supporting a model where the degree of switching to non-functional *ENPP3* isoforms through RTE co-option correlated with disease outcome.

### Loss of tumour cell-intrinsic *CHRNA5* function by *Alu* exonisation

*CHRNA5* (cholinergic receptor nicotinic alpha 5 subunit) encodes the alpha 5 subunit of heteropentameric nicotinic acetylcholine receptor (nAChR) complexes, which initiate signalling cascades upon ligand-gated ion influx [72]. Multiple studies have linked a genetic variant in *CHRNA5* exon 5 (rs16969968) with susceptibility to lung cancer, both through indirect effects on nicotine dependence and smoking behaviour, and through direct tumour cell-intrinsic effects [73–76]. A direct effect of *CHRNA5* expression on tumour cell-intrinsic growth, migration and invasion has been supported by several in vitro studies, although the outcome is likely dependent on the expression pattern of other nAChR subunits expressed in each experimental system [77–80].

**Fig. 7:**
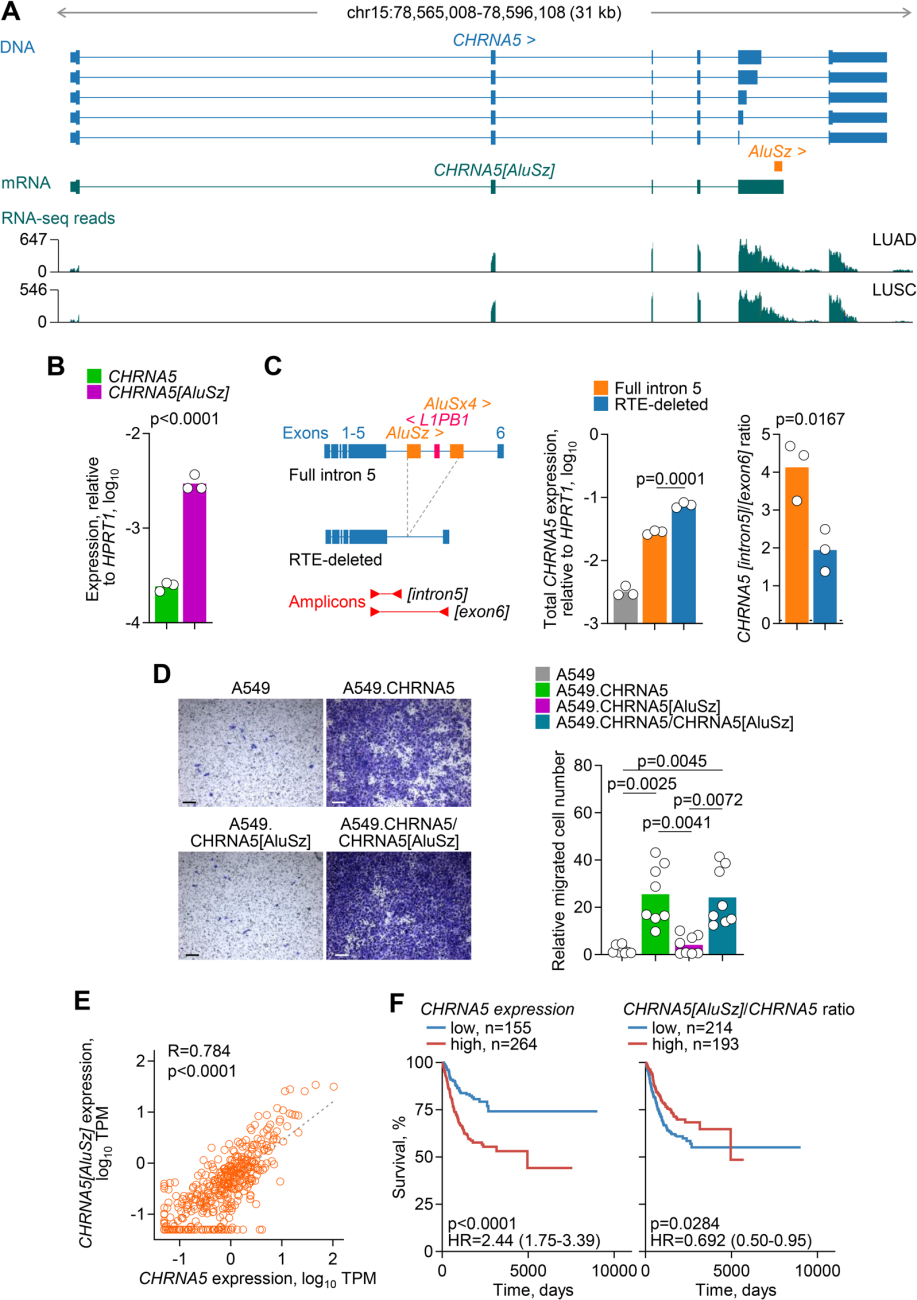
Characterisation of the *CHRNA5[AluSz]* isoform. **A** Gene structure and location of exonised *AluSz*, assembled *CHRNA5[AluSz]* transcript, and RNA-seq traces of 24 combined LUAD and LUSC samples at the *CHRNA5* locus. **B***CHRNA5* and *CHRNA5[AluSz]* expression (assessed by RT-PCR and plotted relatively to *HPRT1* expression) in A549 cells. Symbols represent replicates (*n* = 3) from a single experiment (*p* value calculated with two-tailed Student’s *t*-test). **C***Left*, Schematic representation of *CHRNA5* cDNA minigene constructs retaining only intron 5 either with the reference complement of RTEs (full intron 5) or with the *AluSz* and adjacent *L1PB1* and *AluSx4* elements deleted (RTE-deleted), and of the amplicon used to measure expression. *Right*, Total *CHRNA5* expression (assessed by RT-PCR and plotted relatively to *HPRT1* expression), and ratio of *CHRNA5[intron5]* to *CHRNA5[exon6]* in parental A549 cells or those transfected with either construct. Symbols represent replicates (*n* = 3) from a single experiment (*p* values calculated with two-tailed Student’s *t*-tests between the full intron 5 and RTE-deleted transfections). **D** Representative crystal violet staining of in vitro migrated parental A549 cells and A549 cells expressing the canonical *CHRNA5*, the *CHRNA5[AluSz]* or both isoforms (*left*) (scale bar = 200 µm), and quantitation of the migrated cell number of each genotype (*right*). Symbols represent independent measurements (*n* = 8, 4 fields of view from 2 independent experiments; *p* values calculated with Kruskal–Wallis test with Dunn’s multiple comparisons correction). **E** Correlation of *CHRNA5* and *CHRNA5[AluSz]* expression in LUAD samples (*n* = 419) (*p* value calculated with linear regression). **F** Overall survival of LUAD patients, stratified by *CHRNA5* expression (*left*) or the ratio of *CHRNA5[AluSz]* to *CHRNA5* expression (*right*) (*p* values calculated with log-rank tests)

The regulated use of alternative splice donor sites within exon 5 generates several annotated *CHRNA5* isoforms that all use the canonical terminal exon 6 [81, 82] (Fig. 7A). We have identified a novel transcript, referred to here as *CHRNA5[AluSz]*, which uses an intronic *AluSz* element as alternative terminal exon and polyadenylation site (Fig. 7A). *AluSz* exonisation was confirmed by RT-PCR in HEK293 T, lung adenocarcinoma A549 and oesophageal adenocarcinoma OE19 cells (Additional file 1: Fig. S9), as well as by analysis of long-read RNA-seq data from HEK293 T and A549 cells [22], and oesophageal squamous cell carcinoma (ESCC) TE5 and normal immortalised oesophageal squamous epithelial SHEE cells [21] (Additional file 1: Fig. S10 A). Remarkably, *CHRNA5[AluSz]* appeared to be the dominant isoform in fully transformed A549 and TE5 cells, whereas the balance shifted in favour of the canonical isoform in HEK293 T and non-transformed SHEE cells (Additional file 1: Fig. S10 A). Predominant expression of the *CHRNA5[AluSz]* isoform was also apparent when assessed by RT-qPCR in A549 cells (Fig. 7B) and was also observed in analysis of RNA-seq data from NSCLC and ESCC cell lines in CCLE, whereas several neuroblastoma cell lines, originating from a tissue where *CHRNA5* is physiologically highly expressed, exhibited expression additionally of the canonical isoform (Additional file 1: Fig. S10B). These results implied that, despite not being previously identified, *CHRNA5[AluSz]* is the major isoform expressed particularly in cancer. Consistent, with this notion, both the canonical and the *CHRNA5[AluSz]* isoforms were highly upregulated in several cancer types, compared with the respective normal tissues, with expression of the *CHRNA5[AluSz]* approaching or often exceeding that of the canonical isoform (Additional file 1: Fig. S11).

To examine the effect of the intronic RTEs on *CHRNA5[AluSz]* expression, we tested minigene constructs of *CHRNA5* cDNA retaining only intron 5 with the reference complement of RTEs or with the *AluSz* and adjacent *L1PB1* and *AluSx4* elements deleted (Fig. 7C). Overall *CHRNA5* transcription from either minigene construct transfected into A549 cells exceeded endogenous CHRNA5 expression by at least one order of magnitude, but deletion of intronic RTEs resulted in higher levels of transcription compared with the full intron 5 (Fig. 7C). Moreover, deletion of the intronic RTEs caused a significant shift in the balance of the two isoforms in favour of the canonical isoform terminating in exon 6 (*CHRNA5[exon6]*), although isoforms produced by continued transcription into intron 5 (*CHRNA5[intron5]*) still remained dominant (Fig. 7C). These results suggested that, although not essential, the presence of the specific RTEs in *CHRNA5* intron 5 favours the production of the intronically terminated *CHRNA5* isoform over the canonical.

Given its high expression, we next assessed the potential of the *CHRNA5[AluSz]* isoform to produce a functional protein. At the protein level, *AluSz* exonisation replaces the last 52 amino acids, which are encoded by canonical exon 6 and include the 4 th transmembrane helix of CHRNA5, with as shorter sequence, predicted to remain cytoplasmic (Additional file 1: Fig. S12 A, B). Expression of HA-tagged versions of the canonical CHRNA5 and CHRNA5[AluSz] protein isoforms showed equivalent cell-surface expression in HEK293 T, visualised by immunofluorescence (Additional file 1: Fig. S12 C), indicating efficient translation and plasma membrane trafficking of both. To examine the potential biological activity of the CHRNA5[AluSz] protein, we stably expressed either isoform in A549 cells (Additional file 1: Fig. S13). Neither isoform significantly affected the in vitro growth rate of A549 cells (Additional file 1: Fig. S14). Consistent with prior reports [79, 80], expression of the canonical isoform dramatically enhanced migration of A549 cells, whereas expression of CHRNA5[AluSz] had no apparent effect (Fig. 7D). These results suggested that the loss of the last transmembrane helix resulted in a non-functional CHRNA5 isoform. Interestingly, acquired mutations resulting in an identical truncation of CHRNA6 have been found responsible for evolved insect resistance to insecticides [83], further highlighting the essential function of the last transmembrane helix. To test whether incorporation of the truncated CHRNA5[AluSz] protein into heteropentamers could potentially interfere with the function of the canonical, we co-expressed both isoforms in A549 cells (Additional file 1: Fig. S13). In this setting, cell migration was still significantly enhanced in doubly-expressing A549 cells, at levels comparable with those of A549 cells expressing the canonical isoform only (Fig. 7D). These findings argued against a negative effect of CHRNA5[AluSz], in agreement with the lack of ligand binding by the CHRNA5 subunit [72].

Collectively, these results indicated that the switch to *CHRNA5[AluSz]* expression we observed in cancer would severely compromise the levels of canonical *CHRNA5* that would otherwise be produced and, in turn, reduce the pro-tumour effects of *CHRNA5* expression. A similar effect could also be achieved by alternative splicing within exon 5, causing a frame-shift in the translation of the exon 6 and producing a similarly truncated isoform (NCBI ID: NP_001382100). Although other alternative, non-functional isoforms can be detected [81, 82], the *CHRNA5[AluSz]* appears to be the dominant isoform (Additional file 1: Fig. S10). Expression of *CHRNA5[AluSz]* was significantly correlated with that of the canonical isoform in LUAD samples, although their ratio varied considerably among individual cases (Fig. 7E). In agreement with a pro-tumour role, high overall *CHRNA5* transcription was associated with worse prognosis in LUAD (Fig. 7F). However, a higher fraction of *CHRNA5* transcription diverted to the *CHRNA5[AluSz]* isoform was associated with better prognosis in the same cohort (Fig. 7F), suggesting a protective effect of a switch to the non-functional isoform.

### Evolutionary consideration of RTEs affecting tumour-promoting genes

**Fig. 8:**
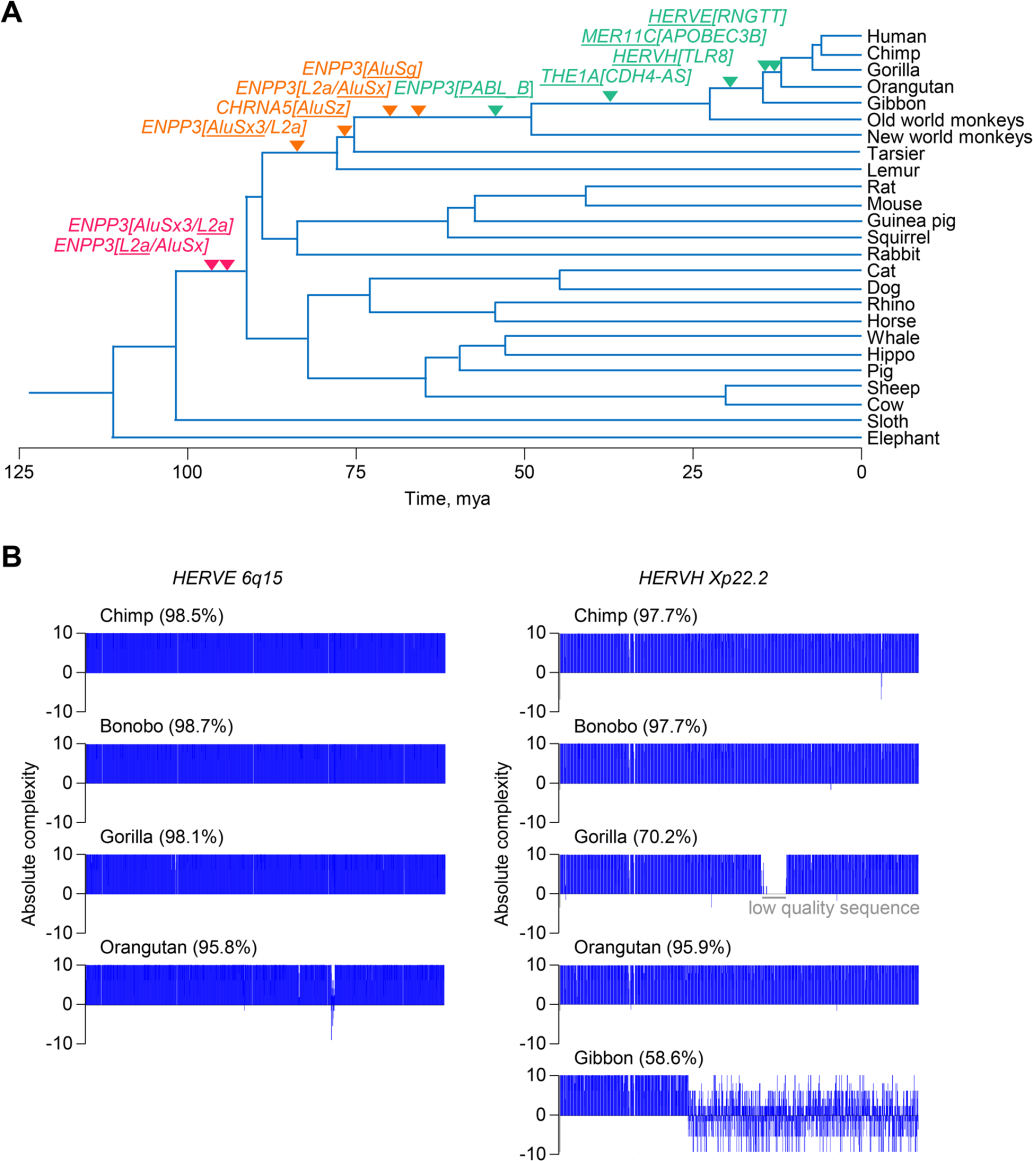
Evolutionary conservation of RTEs affecting tumour-promoting genes. **A** Estimation of the evolutionary age of the specific RTE integrations (underlined) in the indicated genes, based on genomic sequence alignments. **B** Nucleotide sequence homology over the proviral length of the *HERVE 6q15* (8.4 Kb) and *HERVH Xp22.2* (5.3 Kb) integrations between humans and the indicated species. Negative values of absolute complexity denote dissimilarity and numbers in brackets represent percent sequence identity. The gap in the gorilla *HERVH Xp22.2* sequence is due to low-quality sequence

The individual examples of RTE effects on tumour-promoting genes indicated distinct potential mechanisms underlying the transcriptional activation and utilisation of the RTEs involved. In the cases of *ENPP3* and *CHRNA5*, transcription of which is strongly upregulated in tumours, it was possible that the epigenetic changes or selection processes that lead to their upregulation are also responsible for transcriptional utilisation of embedded RTEs that are subsequently exonised, partially counteracting the upregulation of the functional gene isoforms. The RTEs involved in these cases are short, intronic repeats of the *Alu* and *L2a* families, that appear to have integrated into the *ENPP3* and *CHRNA5* loci between 75 and 100 million years ago (Fig. 8A), in line with the evolutionary age of the respective families [1], and have been preserved in the human genome.

In contrast, other loci, including *RNGTT*, the *TLR7* and *TLR8* loci, and *CDH4*, are constitutively or inducibly expressed also in healthy tissue and their expression in tumours is downregulated by the independent transcriptional activation of nearby RTEs. The RTEs involved in these cases are LTR elements, integrated more recently in evolutionary history (Fig. 8A), that have retained the ability to initiate transcription. The *HERVE 6q15* and *HERVH Xp22.2* loci, regulating *RNGTT* and the *TLR7* and *TLR8* loci, respectively, represent complete or near complete proviruses that are well conserved since their integration into the genome of primate ancestors (Fig. 8B). Indeed, sequence identity of the *HERVE 6q15* provirus between humans and the other higher primates, in which it is found, is comparable or higher than that expected by estimates of the rate of synonymous mutations in coding genes [84], whereas the slightly older *HERVH Xp22.2* provirus is relatively less conserved and has sustained a large deletion in the gibbon genome (Fig. 8B). While these examples do not allow broad generalisation, they do highlight the potential for evolutionary conserved RTEs to participate in the negative regulation of tumour-promoting genes.

## Discussion

Evolutionary selection against deleterious effects is constantly depleting the germline of RTE integrations that pose a threat to nearby genes [85]. Nevertheless, numerous recent germline RTE integrations can adversely affect gene function, when the mechanisms that normally prevent their transcription and inclusion in gene transcripts fail. Our data indicate that transcriptional activation of RTEs causes widespread disruption of the transcriptional programme in cancer. Although they would be expected to be counterselected during tumour evolution, we identified several exemplar cases where transcriptional activation of embedded RTEs disrupts the function of a tumour-promoting or essential gene. Our assembly was guided by the current reference genome (GRCh38/hg38), which may be incomplete both in terms of sequence and RTE annotation. The recent release of the single haploid T2 T-CHM13 assembly [32] completed sequence gaps and uncovered thousands of additional RTE integrations [86]. With greater appreciation of the degree of human genetic diversity, including in RTE content, more cases of RTE effects will be expected to be captured in future studies. The identification of such cases also offers the possibility of developing therapeutic interventions aiming to augment the negative impact of RTEs on the function of nearby tumour-promoting genes, while sparing an essential function of the same genes in non-transformed cells, where these RTEs are not activated.

Transcriptionally activated RTEs appear to disrupt the function of adjacent protein-coding genes by two main mechanisms. The first is reduction in the transcription of the protein-coding isoform by RTE-initiated antisense transcription, as exemplified here by *RNGTT*, *CDH4*, *TLR7* and *APOBEC3B*. Antisense transcription has long been recognised as a mechanism of gene regulation more broadly [87]. Furthermore, RTEs have also been implicated in the initiation of *cis* antisense transcripts that may regulate gene expression under physiological conditions [88]. Of note, RTE integrations driving *cis* natural antisense transcripts are enriched near the 3′ UTR of genes and belong to relatively older *L2* and *MIR* subfamilies of non-LTR elements, implying they have been selected during evolution [88]. In contrast, RTEs identified here as regulators of cancer-promoting genes are primarily intergenic or intronic integrations of HERVs and other LTR elements, suggesting that the transcriptional activation of otherwise suppressed RTEs may extend regulation by antisense transcription to a new set of genes specifically in cancer.

The second mechanism by which transcriptionally activated RTEs can disrupt gene function is a switch to the production of non-functional isoforms by RTE-exonisation and alternative splicing. Switch to a non-functional isoform can be at the expense of the canonical protein-coding isoform, with mutually exclusive expression of the two. However, non-functional RTE-exonising isoforms may also be expressed proportionally with the canonical, yet considerably reduce the functional output the gene would otherwise produce. Such an effect on gene function would still be strong, particularly when the non-functional isoform becomes the dominant isoform, as in the case of *CHRNA5[AluSz]*. The switch to non-functional isoforms appears to involve younger RTEs of the *Alu* and *L1* subfamilies, the transcriptional utilisation of which is shared by diverse cancer types. A cancer-specific switch to non-functional isoforms may also explain the previously noted poor correlation between abundance of RNA transcripts, the quantitation of which often ignores the functional potential, and protein levels encoded from at least some genes in cancer [89].

Widespread RTE-mediated loss of function of tumour-promoting genes, as suggested by our findings, is seemingly at odds with the expected effect on tumour fitness that would disadvantage such events during tumour evolution, but may be further supported by recent evidence. A hypoxia-responsive *LTR12B* RTE has been reported to act as a cryptic promoter of an alternative isoform of *POU5 F1*, encoding the pluripotency transcription factor OCT4, producing a likely non-functional version of this tumour-promoting protein in renal cell carcinoma [90]. Similarly, antisense transcription has been reported to regulate levels of the E3 ubiquitin ligase HECTD2, which would otherwise exert a clear tumour-promoting effect in melanoma [91].

Transcriptional activation of intronic RTEs has also been linked with incomplete mRNA splicing, which reduces levels of fully spliced, functional mRNA isoforms and, consequently, tumour cell fitness [92]. Although prior examples in cancer may be limited, similar events have been reported to affect gene function also in physiological conditions. For example, a truncated, non-functional form of ACE2 is produced during infection or inflammation by an IFN-responsive *MIRb* element, acting as an alternative promoter [93]. The use of an intronic *L2a* element as an alternative terminal exon creates a *CD274* isoform that encodes a soluble version of PD-L1, which not only lacks suppressive activity, but also antagonises the immunosuppressive, membrane-bound, canonical PD-L1 [94]. Similarly, the use of an intronic *Alu* element creates an isoform of *IFNAR2*, encoding a truncated version of the type I IFN receptor subunit 2, acting as a decoy receptor [95].

Collectively, these findings underscore the mutagenic potential of RTE insertions, which may be higher than previously appreciated and further enhanced in cancer by their release from epigenetic control. Dysregulation of RTEs in cancer is considered to serve as a warning signal for the emergence of transformed cells. Transcriptional activation of RTEs creates immunogenic ligands that are recognised by innate immune sensors and adaptive antigen receptors, thereby contributing to tumour immunogenicity and immune control [96, 97]. A potential effect of transcriptionally activated RTEs on the function of tumour-promoting or essential genes may represent an additional barrier to transformation. Similar to the immunogenic functions of transcriptionally activated RTEs, disruption of the cancer transcriptional programme would be subject to counterselection during evolution of individual tumours, but it may be positively selected during the evolution of the host species. Whereas the evolution of new function from RTE exaptation, particularly their utilisation in functional proteins, is thought to be a slow evolutionary process [98], the regulation of adjacent gene function by co-option of transcriptionally metastable RTE integrations may evolve faster.

Several of the genes affected by transcriptional activation of RTEs are known to exert strong cell-intrinsic pro-tumour effects. Considered in isolation, this finding would support a potential anti-tumour role for RTE dysregulation through the disruption of the function of those genes. However, there are a number of confounding factors that increase the complexity of these effects.

Some of the affected genes are pleiotropic, with both tumour cell-intrinsic effects and effects on the immune or stroma microenvironment that can indirectly influence tumour grown. Direct and indirect effects of an affected gene can synergise to promote tumour growth. For example, HECTD2 drives tumour cell-intrinsic proliferation of melanoma cells, as well as the production of immunosuppressive mediators [91], whereas ectopic expression of *CALB1* prevents senescence of squamous lung carcinoma cells, but also prevents pro-tumour recruitment of neutrophils by chemokines that would otherwise be secreted as part of the senescence-associated secretory phenotype [13]. Similarly, ENPP3 promotes cell-intrinsic growth and migration of renal cell carcinoma cells [67, 71], but also regulates the availability of STING ligands for immune cells [70], and given its central role in RNA capping, *RNGTT* has the potential to affect many other genes with indirect effects on tumour growth.

Moreover, while the function of a gene may be clearly pro-tumour in the context of an established tumour or cell line, it may play a different role at a different stage during cancer initiation and progression. It may also be the case that the relative fitness cost incurred by RTE-mediated disruption of pro-tumour gene function is a late event in tumour progression, by which time clonal competition between tumour cells has taken place. It may also be that such fitness costs are an unavoidable consequence of global RTE activation during tumour evolution, but offset by gains in tumour-promoting functions resulting from the same underlying epigenetic changes, so that the net effect on tumour growth is positive, and the effect on each gene has to be considered in the context of all other changes. Lastly, an overall negative effect on tumour cell-intrinsic growth caused of RTE-mediated disruption of the cancer transcriptional programme may still benefit tumours by restraining the exponential growth of late-stage tumours that would otherwise outrun or outpace available resources.

## Conclusions

In summary, we have characterised specific cases of tumour-promoting genes, the function of which is disrupted by RTE integrations that are activated during cellular transformation. Such RTEs can therefore sense the epigenetic and transcriptional alterations in transformed cells and potentially act to protect against cancer. Regardless of the ultimate effect on tumour growth, the identification of metastable RTE integrations able to disrupt gene function in cancer deepens our understanding of tumour evolution and offers opportunities for intervention.

## Supplementary Information

Supplementary Material 1. Additional file 1: Fig. S1. *HERVE 6q15* responsiveness to hypoxia. Fig. S2. Essential function of *RNGTT* in cancer cell lines. Fig. S3. CRISPR/Cas9-mediated deletion of the *HERVE 6q15* provirus. Fig. S4. Correlation of *RNGTT* and *HERVE 6q15* expression with survival in KIRC. Fig. S5. *HERVH Xp22.2* expression across cancers and correlation with *TLR7*, *TLR8* and *PRPS2* expression in LUAD. Fig. S6. *APOBEC3B-AS1* expression across cancers and normal tissues. Fig. S7. Cancer specificity of *ENNP3*, and overlapping gene and RTE expression in KIRC. Fig. S8. Instability of the ENPP3[L2a/AluSx] protein product. Fig. S9. PCR validation of the *CHRNA5[AluSz]* isoform. Fig. S10. Balance of *CHRNA5* isoform expression in cancer cell lines. Fig. S11. *CHRNA5* and *CHRNA5[AluSz]* expression across cancers and normal tissues. Fig. S12. Sequence and expression of the CHRNA5[AluSz] protein. Fig. S13. Establishment of A549 cells expressing *CHRNA5* and *CHRNA5[AluSz]*. Fig. S14. Effect of *CHRNA5* and *CHRNA5[AluSz]* expression on A549 in vitro growth.

## Data Availability

The RNA-seq data generated in this study have been deposited at the EMBL-EBI repository (E-MTAB-14514).

## Abbreviations

APOBEC3: Apolipoprotein B editing complex 3
CCLE: Cancer Cell Line Encyclopedia
CDH4: Cadherin 4
cGAMP: 2′3′-Cyclic guanosine monophosphate
CHRNA5: Cholinergic receptor nicotinic alpha 5 subunit
COAD: Colon adenocarcinoma
EAC: Oesophageal adenocarcinoma
ENPP3: Ectonucleotide pyrophosphatase/phosphodiesterase 3
ESCC: Oesophageal squamous cell carcinoma
GFP: Green fluorescent protein
GTEx: The Genotype-Tissue Expression
HA: Hemagglutinin
HERV: Human endogenous retrovirus
IFN: Interferon
KIRC: Kidney renal clear cell carcinoma
LTR: Long terminal repeat
LUAD: Lung adenocarcinoma
LUSC: Lung squamous cell carcinoma
MESO: Mesothelioma
nAChR: Nicotinic acetylcholine receptor
NSCLC: Non-small cell lung carcinomas
OV: Ovarian serous cystadenocarcinoma
PCR: Polymerase chain reaction
PRPS2: Phosphoribosyl pyrophosphate synthetase 2
RNA-seq: RNA-sequencing
RNGTT: RNA guanylyltransferase and 5′-phosphatase
RTEs: Retrotransposable elements
RT-qPCR: Reverse transcriptase-based quantitative PCR
SKCM: Skin cutaneous melanoma
STING: Stimulator of interferon genes
TCGA: The Cancer Genome Atlas Program
TGCT: Testicular germ cell tumours
TLR: Toll-like receptor
TPM: Transcripts per million
VHL: Von Hippel-Lindau
