# Supplementary material for: Retroelement co-option disrupts the cancer transcriptional programme
Source: Genome Med. 2025 May 7;17:48. doi: 10.1186/s13073-025-01479-9 (PMC12060413; doi:10.1186/s13073-025-01479-9)
Supplement: Supplementary file 1 — Supplementary Material 1. Additional file 1: Fig. S1. HERVE 6q15 responsiveness to hypoxia. Fig. S2. Essential function of RNGTT in cancer cell lines. Fig. S3. CRISPR/Cas9-mediated deletion of the HERVE 6q15 provirus. Fig. S4. Correlation of RNGTT and HERVE 6q15 expression with survival in KIRC. Fig. S5. HERVH Xp22.2 expression across cancers and correlation with TLR7, TLR8 and PRPS2 expression in LUAD. Fig. S6. APOBEC3B-AS1 expression across cancers and normal tissues. Fig. S7. Cancer specificity of ENNP3, and overlapping gene and RTE expression in KIRC. Fig. S8. Instability of the ENPP3[L2a/AluSx] protein product. Fig. S9. PCR validation of the CHRNA5[AluSz] isoform. Fig. S10. Balance of CHRNA5 isoform expression in cancer cell lines. Fig. S11. CHRNA5 and CHRNA5[AluSz] expression across cancers and normal tissues. Fig. S12. Sequence and expression of the CHRNA5[AluSz] protein. Fig. S13. Establishment of A549 cells expressing CHRNA5 and CHRNA5[AluSz]. Fig. S14. Effect of CHRNA5 and CHRNA5[AluSz] expression on A549 in vitro growth. [file 13073_2025_1479_MOESM1_ESM.pdf]

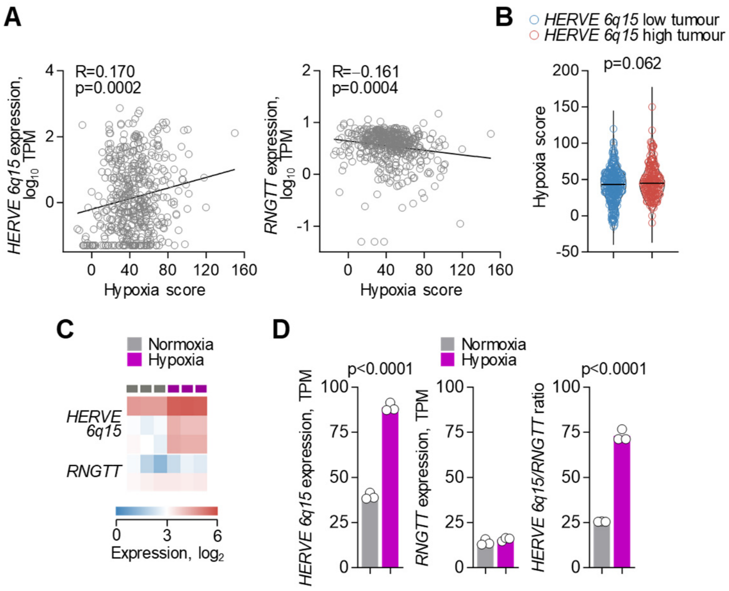

Fig. S1. *HERVE 6q15* responsiveness to hypoxia. (A) *HERVE 6q15* and *RNGTT* expression (TPM) in KIRC samples (n=485) according to their hypoxia scores (p values calculated with linear regression). **(B)** Hypoxia score in KIRC samples with low (n=285) and high (n=200) *HERVE 6q15* expression (p value calculated with Mann-Whitney test). **(C)** Expression of transcripts overlapping *HERVE 6q15* or the canonical *RNGTT* in RNA-seq data (GSE120887) from VHL-sufficient RCC4 cells grown in normoxic or hypoxic conditions [20]. **(D)** *HERVE 6q15* and *RNGTT* expression (TPM), and ratio of *HERVE 6q15* to *RNGTT* expression in the same cells as in c (p values calculated with two-tailed Student's t-tests).

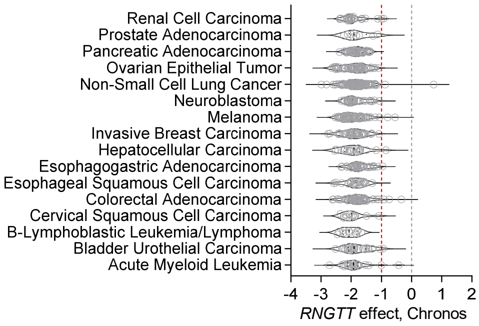

Fig. S2. Essential function of *RNGTT* in cancer cell lines. Effect of *RNGTT* deletion (Chronos score) on the growth of cancer cell lines of the indicated origin. Red dashed line represents the threshold for a significant effect. Data downloaded from the Dependency Map (DepMap) portal [35].

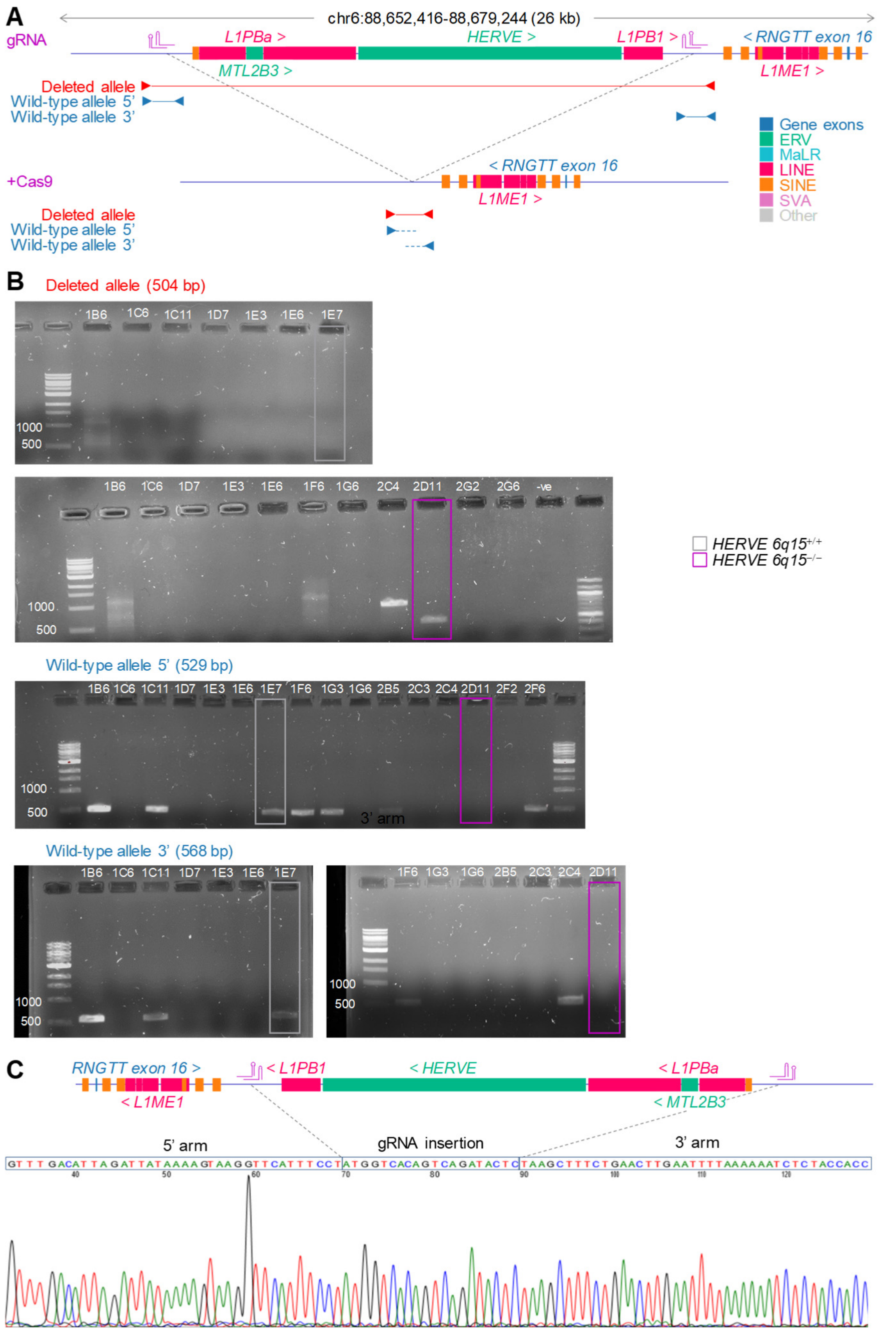

Fig. S3. CRISPR/Cas9-mediated deletion of the *HERVE 6q15* provirus. (A) RTE content in the intronic region of *HERVE 6q15* integration, position of guide RNA (gRNA) molecules, and position of the PCR primers used for the detection of the wild-type and deleted alleles. **(B)** Gel electrophoresis of amplicons from A498 clones with wild-type or deleted alleles. **(C)** Sanger sequencing of PCR amplicon of the deleted allele in *HERVE 6q15*$^{-/-}$ clone 2D11. Also noted is the insertion of one gRNA sequence in site of the deletion.

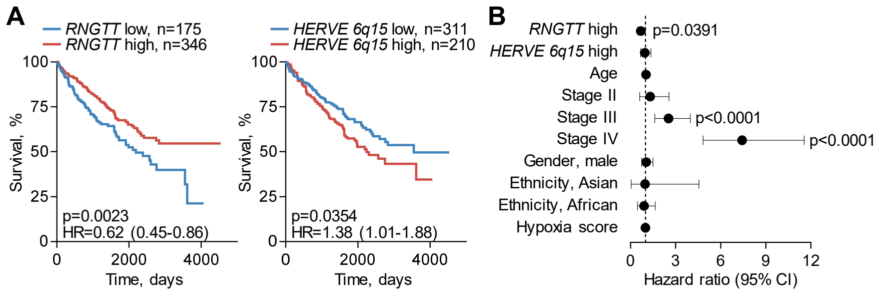

Fig. S4. Correlation of *RNGTT* and *HERVE 6q15* expression with survival in KIRC. (A) Overall survival of KIRC patients, stratified by expression levels of *RNGTT* (*left*) or *HERVE 6q15* (*right*) (p values calculated with log-rank tests). **(B)** Overall survival hazard ratios (HRs) for the indicated variables in KIRC patients (*RNGTT* high n=346, reference low n=175; *HERVE 6q15* high n=210, reference low n=311; Age n=521; Stage II n=56, III n=123, IV n=82, reference I n=257; Gender, male n=338, reference female n=183; Ethnicity, Asian n=8, African n=55, reference White n=450; Hypoxia score n=470). Error bars represent 95% confidence intervals (CIs) (p values calculated with Cox proportional hazards regression).

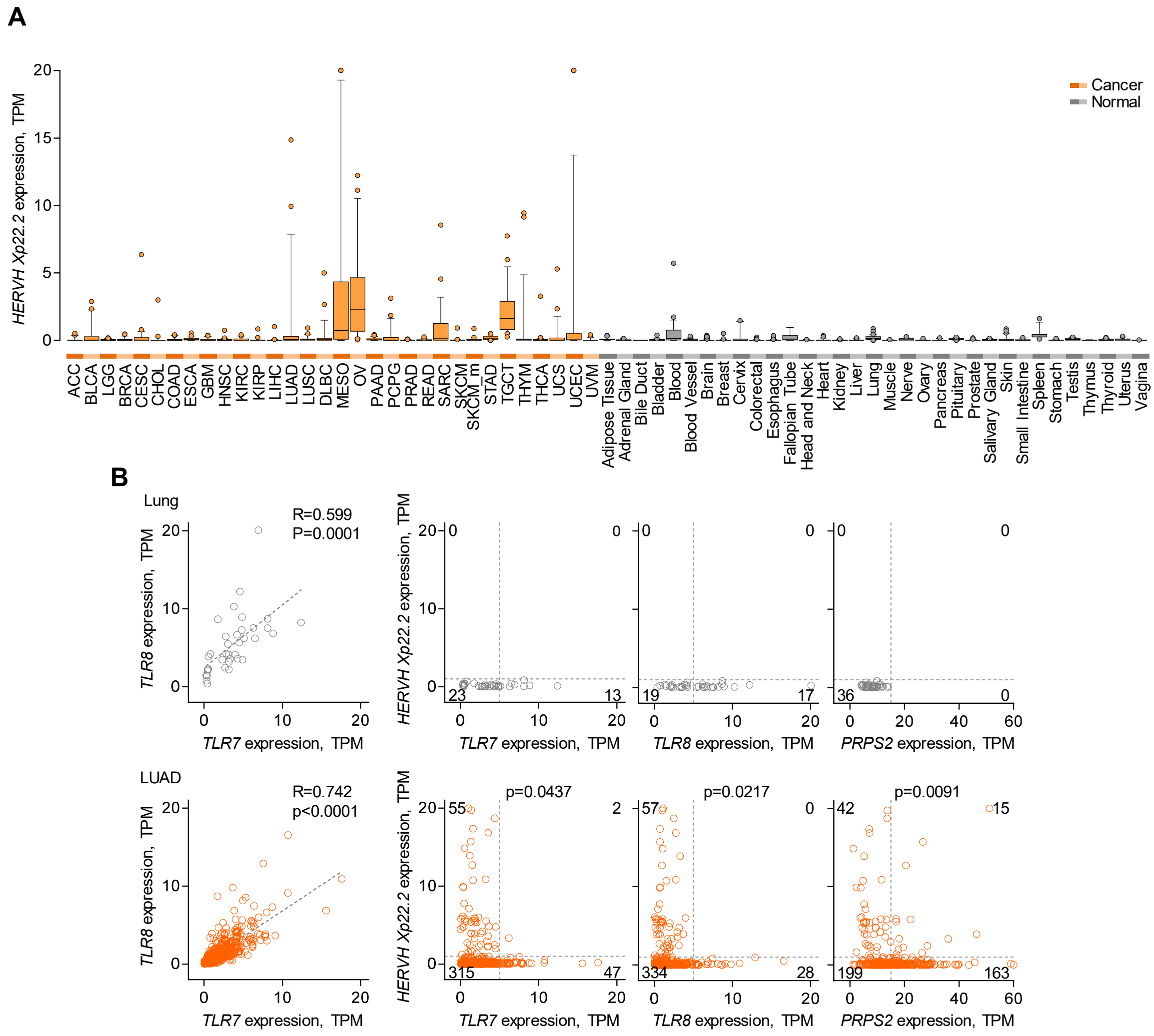

Fig. S5. *HERVH Xp22.2* expression across cancers and correlation with *TLR7*, *TLR8* and *PRPS2* expression in LUAD. (A) *HERVH Xp22.2* expression (TPM) in the indicated TCGA cancer types (n = 24 per type) and in normal tissues from GTEx (n=2-156 per tissue type). Box plots denote median value and quartiles, whiskers denote 1.5x the interquartile range, and individual points denote outliers. Values are capped at 20 TPM. **(B)** Correlation of *HERVH Xp22.2*, *TLR7*, *TLR8* and *PRPS2* expression (TPM) in normal lung tissue (n=36) or LUAD samples (n=419) (p values calculated with linear regression for positive correlations, and with Fisher's exact tests for negative correlations using the numbers in the quadrants).

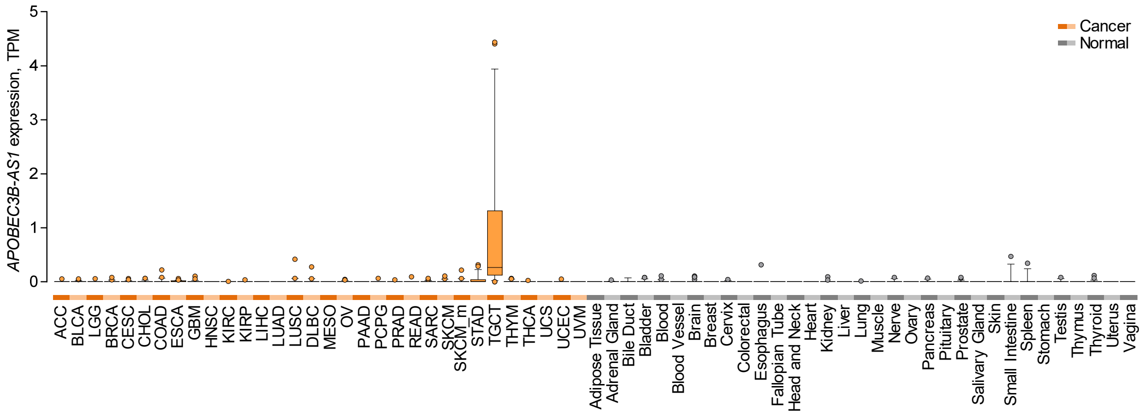

Fig. S6. *APOBEC3B-AS1* expression across cancers and normal tissues. *APOBEC3B-AS1* expression (TPM) in the indicated TCGA cancer types (n = 24 per type) and in normal tissues from GTEx (n = 2-156 per tissue type). Box plots denote median value and quartiles, whiskers denote 1.5x the interquartile range, and individual points denote outliers.

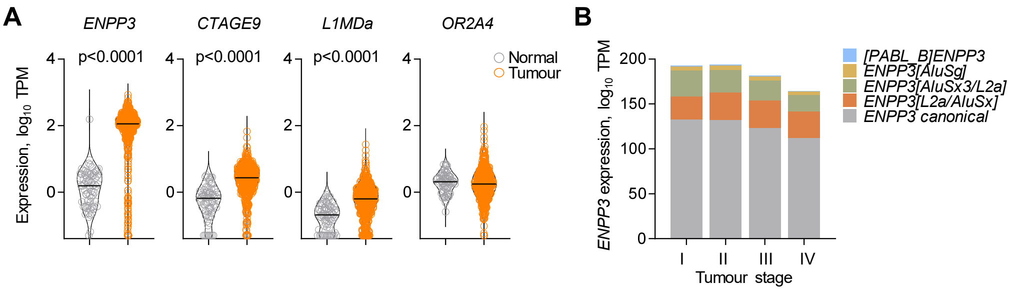

Fig. S7. Cancer specificity of *ENNP3*, and overlapping gene and RTE expression in KIRC. (A) Expression of canonical *ENNP3* or overlapping genes and RTEs in normal kidney tissue (n=71) and KIRC samples (n=538) (p values calculated with Mann-Whitney tests). **(B)** *ENPP3* isoform expression (TPM) in KIRC samples according to tumour stage (I n=271, II n=59, III n=123, IV n=82).

A

```
                   1                                                                                                                     120
                   |          Transmembrane helix                                                                                          |
          ENPP3    MESTLTLATEQPVKKNTLKKYKIACIVLLALLVIMSLGLGLGLGLRKLEKQGSCRKKCFDASFRGLENCRCDVACKDRGDCCWDFEDTCVESTRIWMCNKFRCGETRLEASLCSCSDDCL
ENPP3[L2a/AluSx]   MESTLTLATEQPVKKNTLKKYKIACIVLLALLVIMSLGLGLGLGLRKLEKQGSCRKKCFDASFRGLENCRCDVACKDRGDCCWDFEDTCVESTRIWMCNKFRCGETRLEASLCSCSDDCL
                 121                                                                                                                     240
                   |                                                                                 Phosphodiesterase                     |
          ENPP3    QRKDCCADYKSVCQGETSWLEENCDTAQQSQCPEGFDLPPVILFSMDGFRAEYLYTWDTLMPNINKLKTCGIHSKYMRAMYPTKTFPNHYTIVTGLYPESHGIIDNNMYDVNLNKNFSLS
ENPP3[L2a/AluSx]   QRKDCCADYKSVCQGETSWLEENCDTAQQSQCPEGFDLPPVILFSMDGFRAEYLYTWDTLMPNINKLKTCGIHSKYMRAMYPTKTFPNHYTIVTGLYPESHGIIDNNMYDVNLNKNFSLS
                 241                                                                                                                     360
                   |                                                                                                                       |
          ENPP3    SKEQNNPAWWHGQPMWLTAMYQGLKAATYFWPGSEVAINGSFPSIYMPYNGSVPFEERISTLLKWLDLPKAERPRFYTMYFEEPDSSGHAGGPVSARVIKALQVVDHAFGMLMEGLKQRN
ENPP3[L2a/AluSx]   SKEQNNPAWWHGQPMWLTAMYQGLKAATYFWPGSEVAINGSFPSIYMPYNGSVPFEERISTLLKWLDLPKAERPRFYTMYFEEPDSSGHAGGPVSARVIKALQVVDHAFGMLMEGLKQRN
                 361                                                                                                                     480
                   |                                                                                                                       |
          ENPP3    LHNCVNIILLADHGMDQTYCNKMEYMTDYFPRINFFYMYEGPAPRIRAHNIPHDFFSFNSEEIVRNLSCRKPDQHFKPYLTPDLPKRLHYAKNVRIDKVHLFVDQQWLAVRSKSNTNCGG
ENPP3[L2a/AluSx]   LHNCVNIILLADHGMDQTYCNKMEYMTDYFPRINFFYMYEGPAPRIRAHNIPHDFFSFNSEEIVRNLSCRKPDQHFKPYLTPDLPKRLHYAKNVRIDKVHLFVDQQWLAVRFVYLFTYLI
                 481                                                                                                                     600
                   |                                                                                                                       |
          ENPP3    GNHGYNNEFRSMEAIFLAHGPSFKEKTEVEPFENIEVYNLMCDLLRIQPAPNNGTHGSLNHLLKVPFYEPSHAEEVSKFSVCGFANPLPTESLDCFCPHLQNSTQLEQVNQMLNLTQEEI
ENPP3[L2a/AluSx]   MPLITICHYKST-----------------------------------------------------------------------------------------------------------
                 601                                                                                                                     720
                   |                                                                                       Nuclease                        |
          ENPP3    TATVKVNLPFGRPRVLQKNVDHCLLYHREYVSGFGKAMRMPMWSSYTVPQLGDTSPLPPTVPDCLRADVRVPPSESQKCSFYLADKNITHGFLYPPASNRTSDSQYDALITSNLVPMYEE
ENPP3[L2a/AluSx]   -----------------------------------------------------------------------------------------------------------------------
                 720                                                                                                                     840
                   |                                                                                                                       |
          ENPP3    FRKMWDYFHSVLLIKHATERNGVNVVSGPIFDYNYDGHFDAPDEITKHLANTDVPIPTHYFVVLTSCKNKSHTPENCPGWLDVLPFIIPHRPTNVESCPEGKPEALWVEERFTAHIARVR
ENPP3[L2a/AluSx]   -----------------------------------------------------------------------------------------------------------------------
                 841                                  875
                   |                                    |
          ENPP3    DVELLTGLDFYQDKVQPVSEILQLKTYLPTFETTI
ENPP3[L2a/AluSx]   -----------------------------------
```

B

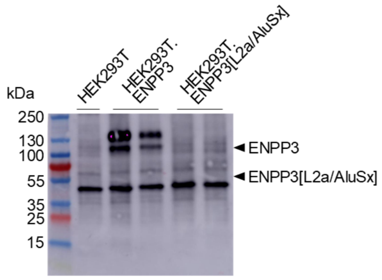

Fig. S8. Instability of the *ENPP3[L2a/AluSx]* protein product. (A) Amino acid sequence alignment of canonical ENPP3 and the ENPP3[L2a/AluSx] isoform. Amino acid residues in red denote the sequence in ENPP3[L2a/AluSx] that replaces the end of the phosphodiesterase and all of the nuclease domain. **(B)** Western blot for ENPP3 in lysates from parental HEK293T cells and cells transduced to express ENPP3 and ENPP3[L2a/AluSx] (HEK293T.ENPP3 and HEK293T.ENPP3[L2a/AluSx], respectively, run in duplicate). Arrows show the theoretical mass of each isoform.

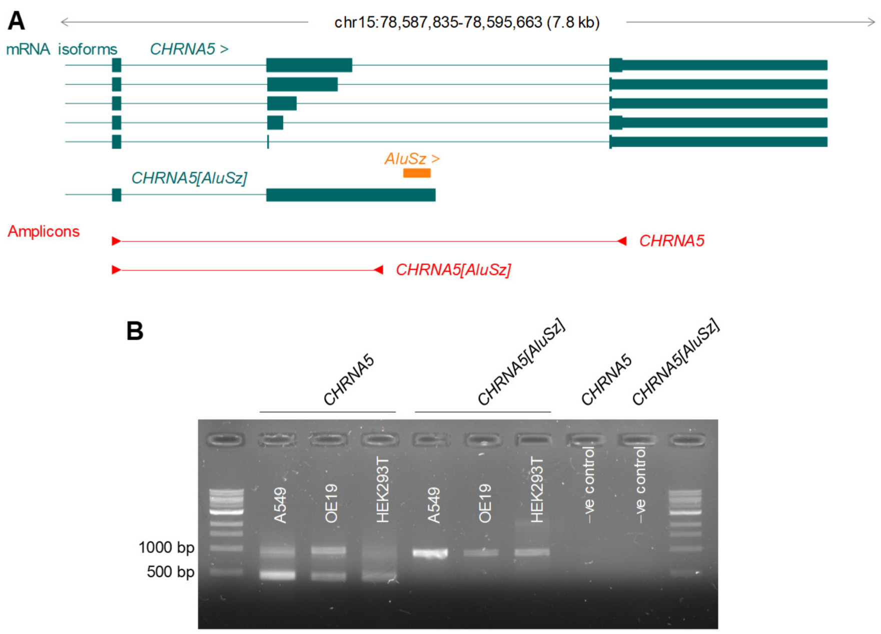

Fig. S9. PCR validation of the *CHRNA5[AluSz]* isoform. (A) Annotated and assembled *CHRNA5* transcripts (exons 4-6 only), location of exonised *AluSz*, and amplicons used for transcript validation. **(B)** Gel electrophoresis of amplicons of *CHRNA5[AluSz]* or canonical *CHRNA5* isoforms amplified from the indicated cell lines. *CHRNA5* amplicons of different sizes correspond to canonical isoforms utilising different splice donor sites in exon 5.

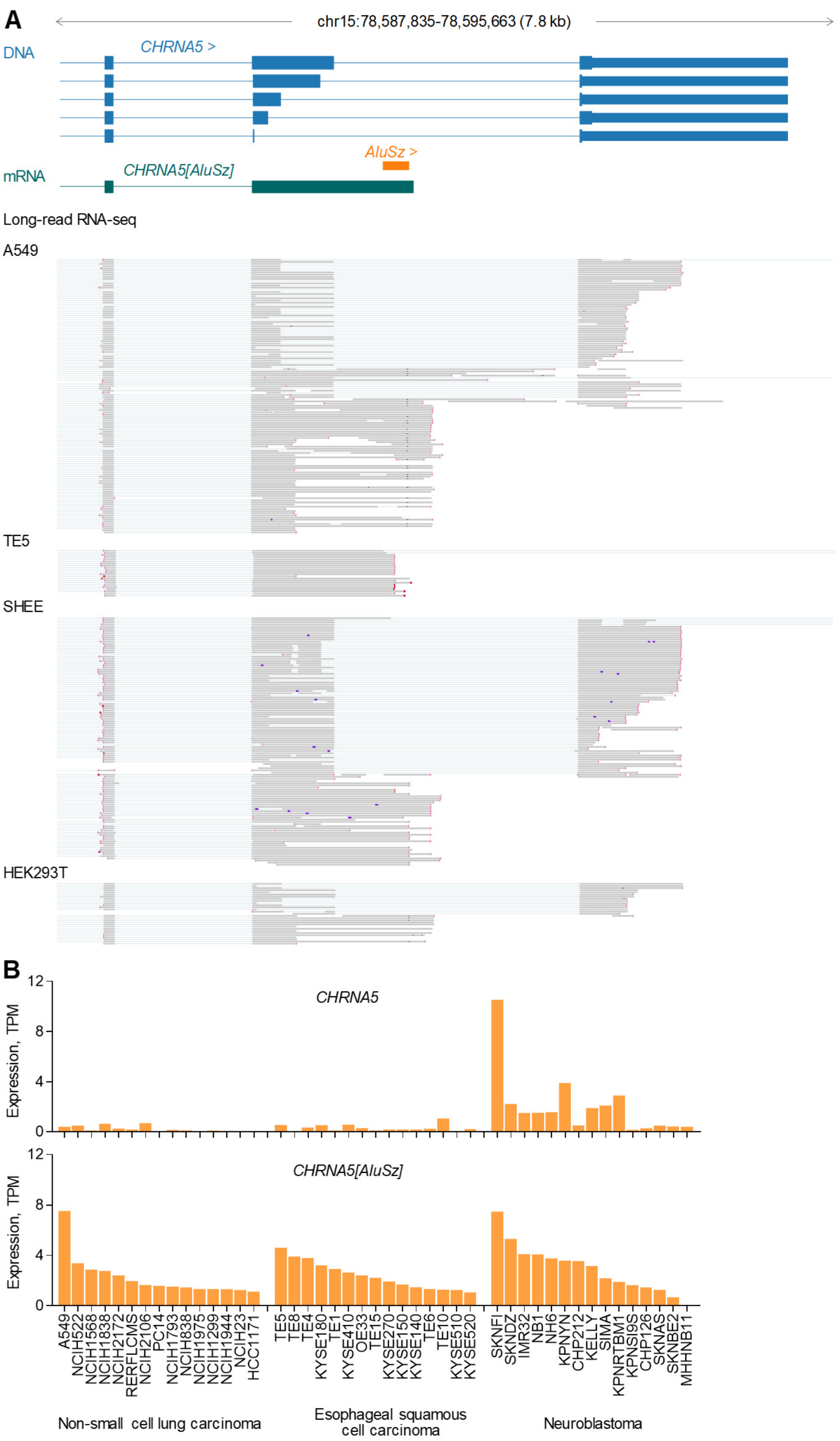

Fig. S10. Balance of *CHRNA5* isoform expression in cancer cell lines. (A) Gene structure (exons 4-6 only), location of exonised *AluSz*, assembled *CHRNA5[AluSz]* transcript, and long-read RNA-seq data from HEK293T and A549 cells [22], and esophageal squamous cell carcinoma TE5 and normal immortalized esophageal squamous epithelial SHEE cells (PRJNA515570) [21]. **(B)** *CHRNA5* and *CHRNA5[AluSz]* isoform expression (TPM) in RNA-seq data from the indicated cell lines in CCLE.

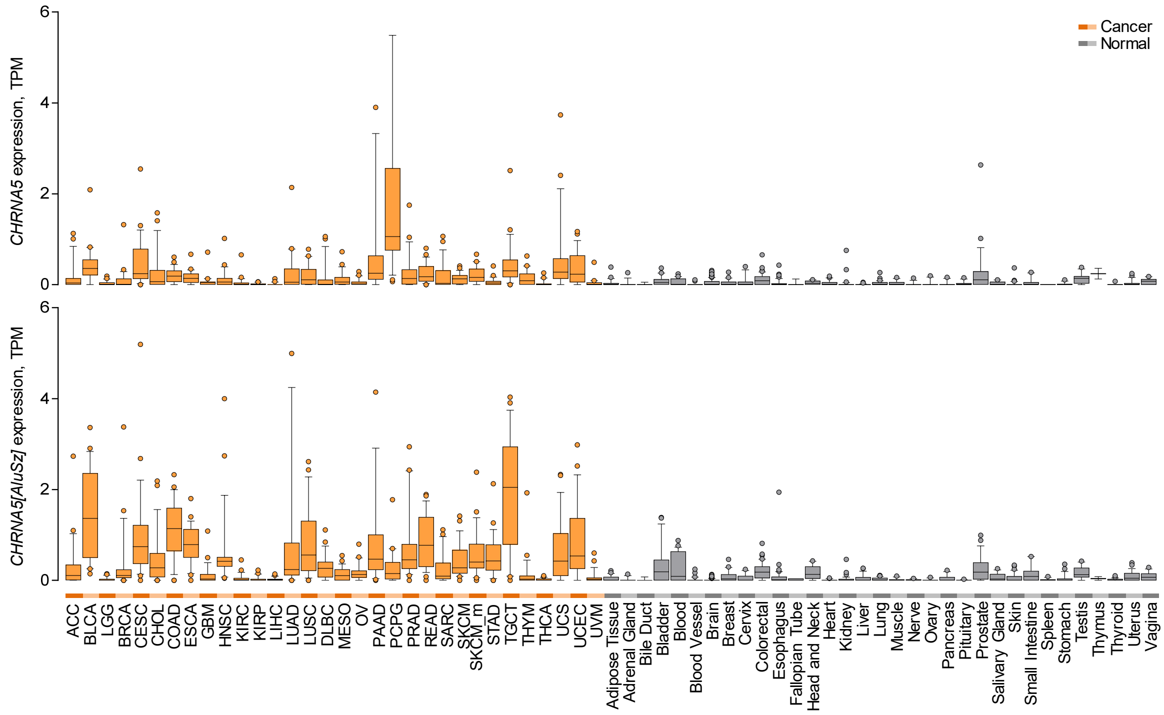

Fig. S11. *CHRNA5* and *CHRNA5[AluSz]* expression across cancers and normal tissues. Canonical *CHRNA5* and *CHRNA5[AluSz]* isoform expression (TPM) in the indicated TCGA cancer types (n = 24 per type) and in normal tissues from GTEx (n = 2-156 per tissue type). For canonical *CHRNA5*, only the isoform with the full-length exon 5, encoding the full-length protein, is included. Box plots denote median value and quartiles, whiskers denote 1.5x the interquartile range, and individual points denote outliers.

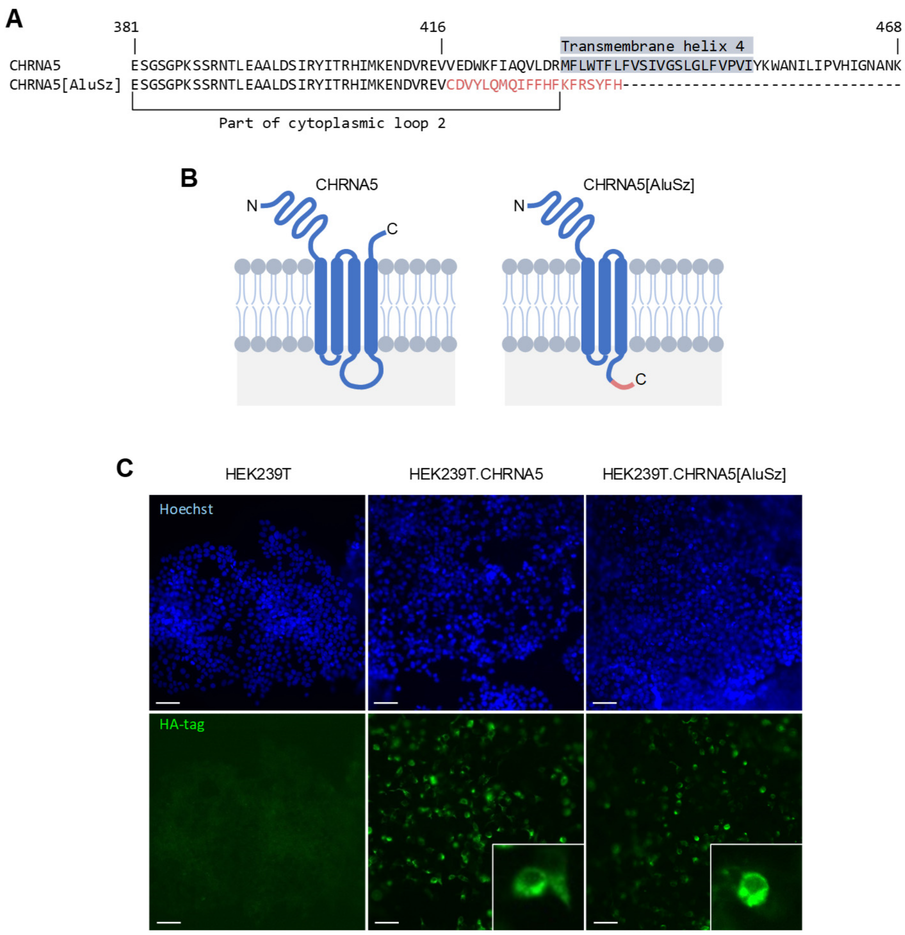

Fig. S12. Sequence and expression of the CHRNA5[AluSz] protein. (A) Amino acid sequence alignment of canonical CHRNA5 (last 88 amino acids only) and the CHRNA5[AluSz] isoform. Amino acid residues in red denote the sequence in CHRNA5[AluSz] that replaces the end of the last transmembrane helix and extracellular C-terminal sequence of the canonical isoform. **(B)** Schematic representation of canonical CHRNA5 and the CHRNA5[AluSz] isoform. **(C)** Immunofluorescence detection of cell surface expression (in non-permeabilised cells) of HA-tagged canonical CHRNA5 and CHRNA5[AluSz] isoforms, overexpressed in HEK293T cells. Images are from a single experiment (scale bar=100µm, insets 5× magnification).

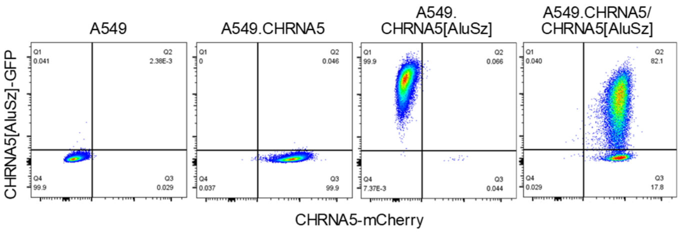

Fig. S13. Establishment of A549 cells expressing *CHRNA5* and *CHRNA5[AluSz]*. Flow cytometric detection of fluorescent reporter expression in parental A549 cells and A549 cells expressing the canonical *CHRNA5* isoform and mCherry reporter, the *CHRNA5[AluSz]* isoform and GFP reporter or both isoforms.

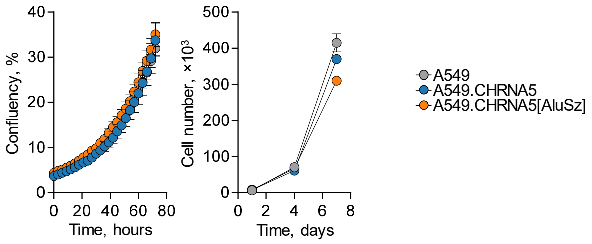

Fig. S14. Effect of *CHRNA5* and *CHRNA5[AluSz]* expression on A549 *in vitro* growth. Mean confluency (±SEM, n=6 from 1 experiment) (*left*) and mean cell number (±SEM, n=2 from 1 experiment) (*right*) of parental A549 cell cultures and those of A549 cells expressing the canonical *CHRNA5* or the *CHRNA5[AluSz]* isoform.
